# Follicle-intrinsic and spatially distinct molecular programs drive follicle rupture and luteinization during ex vivo mammalian ovulation

**DOI:** 10.1038/s42003-024-07074-9

**Published:** 2024-10-23

**Authors:** Emily J. Zaniker, Jiyang Zhang, Daniela Russo, Ruixu Huang, Kristine Suritis, Riley S. Drake, Esther Barlow-Smith, Alex K. Shalek, Teresa K. Woodruff, Shuo Xiao, Brittany A. Goods, Francesca E. Duncan

**Affiliations:** 1 Department of Obstetrics and Gynecology, Feinberg School of Medicine, Northwestern University, Chicago, IL 60611 USA; 2 Department of Pharmacology and Toxicology, Ernest Mario School of Pharmacy, Rutgers University, Piscataway, NJ USA; 3 Institute for Medical Engineering & Science, Department of Chemistry, and Koch Institute for Integrative Cancer Research Massachusetts Institute of Technology, Cambridge, MA 02139 USA; 4 Broad Institute, Harvard University & Massachusetts Institute of Technology, Cambridge, MA 02139 USA; 5 The Ragon Institute of MGH, MIT and Harvard, Cambridge, MA 02139 USA; 6 Thayer School of Engineering, Dartmouth College, Hanover, NH USA; 7 Micrographia Bio, London, UK; 8 Department of Obstetrics and Gynecology, Michigan State University, East Lansing, MI USA

**Keywords:** Reproductive biology, Transcriptomics

## Abstract

During ovulation, the apical wall of the preovulatory follicle breaks down to facilitate gamete release. In parallel, the residual follicle wall differentiates into a progesterone-producing corpus luteum. Disruption of ovulation, whether through contraceptive intervention or infertility, has implications for women’s health. In this study, we harness the power of an ex vivo ovulation model and machine-learning guided microdissection to identify differences between the ruptured and unruptured sides of the follicle wall. We demonstrate that the unruptured side exhibits clear markers of luteinization after ovulation while the ruptured side exhibits cell death signals. RNA-sequencing of individual follicle sides reveals 2099 differentially expressed genes (DEGs) between follicle sides without ovulation induction, and 1673 DEGs 12 h after induction of ovulation. Our model validates molecular patterns consistent with known ovulation biology even though this process occurs in the absence of the ovarian stroma, vasculature, and immune cells. We further identify previously unappreciated pathways including amino acid transport and Jag-Notch signaling on the ruptured side and glycolysis, metal ion processing, and IL-11 signaling on the unruptured side of the follicle. This study yields key insights into follicle-inherent, spatially-defined pathways that underlie follicle rupture, which may further understanding of ovulation physiology and advance women’s health.

## Abstract (web summary)

Molecular profiling of ex vivo ovarian follicles reveals key asymmetric pathways that underlie follicle rupture and luteinization during ovulation

## Introduction

Ovulation involves the coordinated release of the cumulus oocyte complex (COC) from the ovarian follicle, which is essential for human fertility. Altered ovulation resulting from endocrinopathies, ovarian pathologies, iatrogenic dysfunction, idiopathic anovulation, or ovarian aging are all associated with subfertility or infertility^1–4^. In addition, ovulation represents an important biological process that can be targeted for the development of non-hormonal contraceptives by identifying ways to block follicle rupture during ovulation without interfering with hormone production during the luteal phase. Such contraceptives would represent a significant advance given that currently available hormonal contraceptives exhibit side effects ranging from mood changes to increased risk of blood clots, resulting in non-use or mis-use^5,6^. Improving our understanding of ovulation on a molecular level, therefore, would have important implications for human health and fertility.

The functional unit of the ovary is the follicle, which is comprised of the germ cell or oocyte surrounded by somatic granulosa cells that support oocyte growth and ovarian hormone homeostasis. Humans are born with a finite pool of follicles, most of which will undergo atresia and will never produce a mature egg^7–11^. A subset of these follicles will develop into antral stage follicles which are characterized by fluid-filled cavities surrounded by mural granulosa cells that form the follicle wall and cumulus granulosa cells which surround the oocyte^7,12–15^. During follicle growth, preantral follicles are symmetric with respect to the organization of the oocyte and its somatic cells, whereas antral follicles exhibit asymmetry with the COC offset within the follicle^16^. In humans, a complex interplay of pituitary gonadotropin hormones and local ovarian factors lead to selection of one follicle, called the dominant follicle, which will fully mature to the ovulatory stage^17–19^. At the time of ovulation, a surge of luteinizing hormone (LH) from the pituitary triggers the process of ovulation by which the dominant follicle ruptures open to release a COC containing a fertilization-competent gamete^20–25^. During ovulation, prominent asymmetric remodeling occurs throughout as the extracellular matrix (ECM) and cellular layers in a distinct region of the follicle wall adjacent to the ovarian surface epithelium are degraded, and the contralateral side of the follicle signals peripheral angiogenic cell invasion, and vasodilation, as well as contractions of the cells in the follicle periphery^12,18–21,26–47^. These cascades culminate in the rupture and release of the COC. The remainder of the follicle wall opposite the site of rupture then undergoes a process of tissue remodeling and cellular transformation called luteinization, where granulosa and theca cells differentiate or luteinize into progesterone-producing luteal cells^18,20,21^. These cells form a transient endocrine structure called the corpus luteum that supports the hormonal axis of the ovulatory cycle and signals change in the uterine lining to stimulate glandular secretion that supports early embryonic development in the case of pregnancy^21,48,49^. Regression of the corpus luteum following ovulation leads to a decline in progesterone levels, which induces shedding of the endometrial lining of the uterus during menstruation^50–52^.

The coordinated processes of follicle rupture on one side of the follicle and luteinization on the opposite side demonstrate a striking asymmetry that is essential for normal follicle function. On a molecular level, there is increased activity of proteases and gelatinases at the site of rupture to facilitate degradation of the ECM in both the follicle wall and the surrounding ovarian surface epithelial cells. In contrast, there is increased expression of protease inhibitors at the opposite side of the follicle wall to maintain tissue integrity in preparation for luteinization^12,31,32,53,54^. This asymmetry may be driven in part by prostaglandin E2 (PGE2) receptor subtypes and other proteins such as those in the semaphorin 7 A (*Sema7a*) family^32,55^. Ovarian microvascular remodeling and smooth-muscle functions also act asymmetrically within the follicle wall during the periovulatory period. In response to the LH surge, there is a decrease in blood flow on the ruptured side, which drives apoptosis and degradation of the rupture site, and a concomitant increase in blood flow on the unruptured side that supports influx of immune cells and provides a vascular supply for the developing corpus luteum^12,33–39,56–71^. Smooth muscle-like contractions are also spatially restricted to the unruptured side of the follicle wall, which is thought to exert a mechanical force that contributes to follicle rupture and COC release to the ovarian surface where it is ultimately picked up by the fimbria of the oviduct^33–35,39,43–47,72–74^.

Despite the essential role that spatial asymmetry plays in follicle rupture during ovulation, there are no existing studies that characterize the functional outputs and molecular signatures of the distinct cell populations on each side of the follicle wall. A comprehensive profile of the molecular pathways that underlie the distinct phenotypes of the ruptured and unruptured sides is lacking given the technical challenge of separating these specific regions of the follicle in vivo. In this study, we used a mouse model of ex vivo follicle growth and ovulation to model both follicle rupture and luteinization to comprehensively assess the follicle-intrinsic functional and molecular signatures of distinct regions of the follicle wall^75–91^. Importantly, this model recapitulates the hormone signaling, transcription factors, proteolytic genes, and inflammatory pathways that occur during in vivo ovulation, thereby enabling high resolution interrogation of the ovulatory period in a tightly controlled system^78,92^. Specifically, we found spatial differences in the luteinization capacity and several asymmetric pathways within the ovulatory follicle wall, demonstrating that the two sides of the follicle wall are functionally and molecularly distinct.

## Results

### The unruptured and ruptured side of the follicle wall exhibit functional differences following ovulation induction

**Fig. 1:**
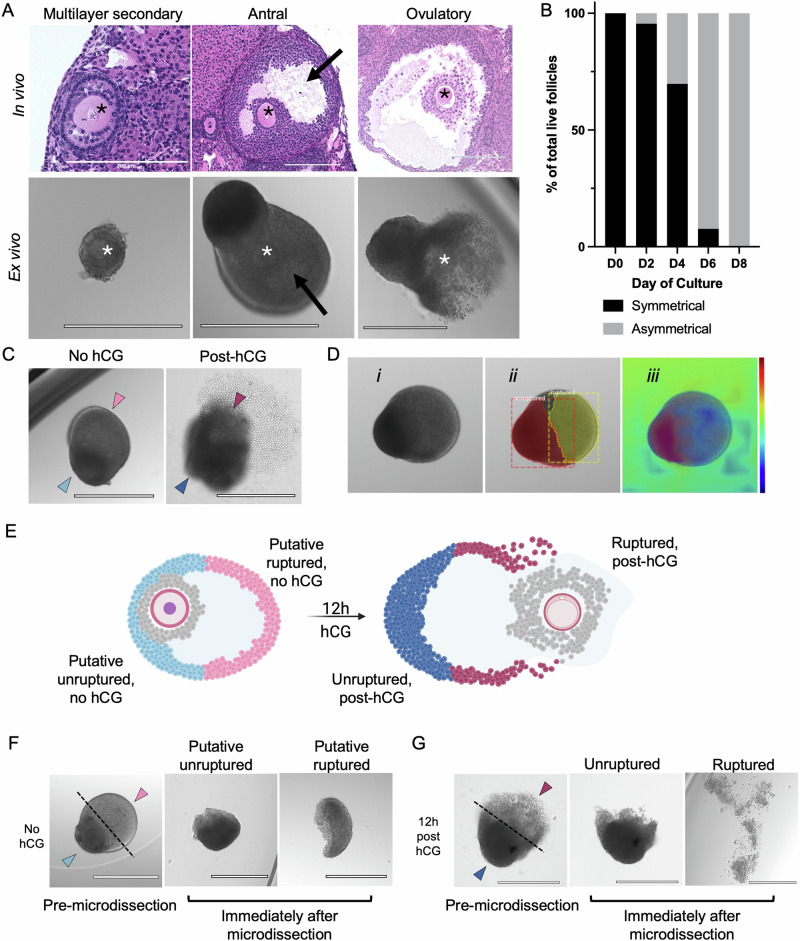
Follicles develop an asymmetric morphology prior to ovulation both in vivo and in vitro, which can be used to predict the site of follicle rupture. **A** During follicle development, follicles grow from a symmetric morphology where the oocyte (asterisk) is surrounded by even layers of granulosa cells, to an asymmetric morphology where the oocyte (asterisk) is surrounded by cumulus cells and a fluid-filled antral cavity forms (arrow). The antral cavity continues to expand and will eventually rupture open to release the cumulus oocyte complex. **B** During ex vivo follicle growth, follicles begin exhibiting signs of asymmetry at around day 4 of culture and become entirely asymmetric by day 8 of culture. *n* = 51 follicles. **C** The site of rupture of follicles grown ex vivo can be reliably predicted based on morphology of the follicle. Rupture occurs in the region of the follicle wall that is expanded due to the antral cavity and is optically lighter in color on brightfield microscopy. **D** Antral stage follicles (*i*) can be subdivided into distinct regions (*ii*) by an object detection model’s prediction of the ruptured (in yellow) follicle region and unruptured (in red) follicle region. (*iii*) The CAM indicates that the model prediction concentrated on differences in cellular density to distinguish between the ruptured and unruptured regions of the follicle. *n* = 92 follicles. **E** Our experimental paradigm refers to these distinct regions as the putative unruptured side and putative ruptured side in the no-hCG condition and as the unruptured and ruptured side in the post-hCG condition. **F** Based on morphologic hallmarks of the putative ruptured and unruptured sides and guidance from the machine learning model, follicles in the no-hCG condition can be microdissected. The COC was removed and excluded so that only the contribution of the follicle was analyzed. Images are from the same follicle, before and after microdissection. The pink arrowhead denotes the putative ruptured side and the light blue arrowhead denotes the putative unruptured side. **G** 12 h post-hCG, follicles were microdissected to separate the unruptured region from the region that has ruptured open during ovulation with the COC removed. Images are from the same follicle, before and after microdissection. The dark pink arrowhead denotes the ruptured side and the dark blue arrowhead denotes the unruptured side.

Ex vivo cultured mouse follicles recapitulated the transition from symmetric early stage preantral follicles to asymmetric antral stage follicles seen in vivo (Fig. 1A–C)^79–86^. Early secondary stage follicles, both in vivo and ex vivo, began as symmetric structures with uniform layers of granulosa cells surrounding the oocyte (Fig. 1A). As follicles matured, they grew asymmetrically and formed a fluid-filled antral cavity that expanded and contributed to the formation of the eventual site of rupture (Fig. 1A). Brightfield images showed that alginate-encapsulated follicles maintained 3D architecture and exhibited asymmetrical growth as early as day 4 of culture, which was driven by formation of the antral cavity and differentiation of granulosa cells into sub-populations of mural granulosa cells that formed the follicle wall and cumulus cells that surround the oocyte (Fig. 1A, B, Supplementary Data 1). The asymmetry in our ex vivo model replicated what was seen in vivo, where the antral cavity formed adjacent to the ovarian surface epithelium and initiated the site of rupture during ovulation, while the remnant of the follicle wall involuted to form the progesterone-producing corpus luteum (Fig. 1A, C). Our ex vivo model, therefore, is a powerful tool to study these follicle sides individually in a controlled model system by manually microdissecting follicles to separate these distinct regions. To harness this asymmetry for our study, we first needed to identify morphologic features that reliably predict the site of rupture (Fig. 1C, D). Using a machine learning model, we demonstrated that a consistent zone of rupture could be distinguished within the region of the follicle wall with reduced optical density resulting from antrum formation (Fig. 1D). Regions of reduced optical density, identified by heat maps in the machine learning model, was used to guide decisions on microdissection to separate the putative ruptured and putative unruptured sides with the COC removed (Fig. 1D–F). In the post-hCG condition, follicles were microdissected to separate the actual site of rupture from the unruptured side of the follicle wall and the COC was removed (Fig. 1E, G).

**Fig. 2:**
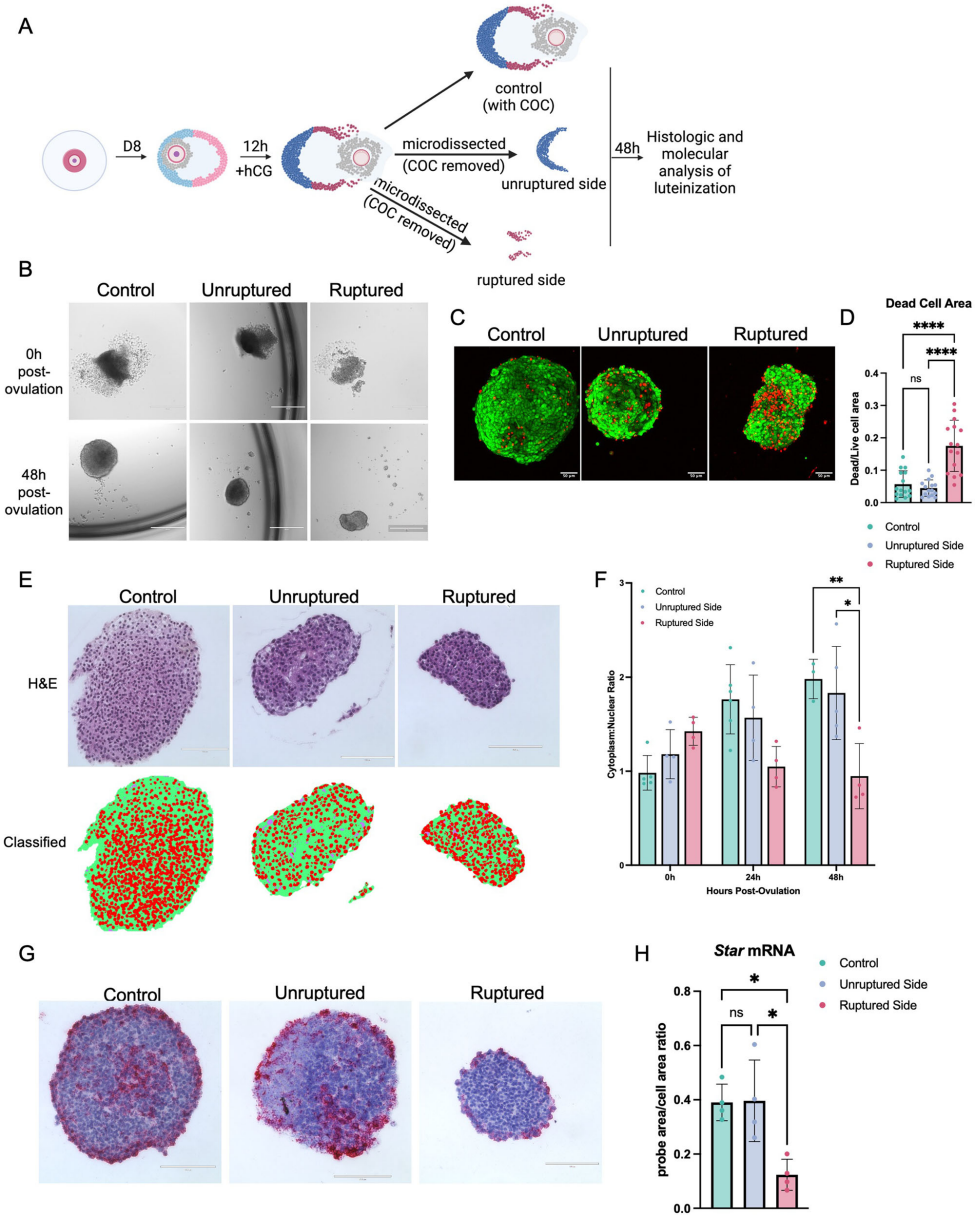
The unruptured side of the follicle wall exhibits key signs of luteinization 48 h post-ovulation, while the ruptured side exhibits signs of cell death during the same period. **A** In our experimental model, we culture follicles in alginate for 8 days until they reach the terminal antral stage. We then remove the follicles from alginate and expose them to media enriched in hCG for 12 h to induce ovulation. Afterwards, follicles were microdissected to separate the unruptured side and the unruptured side with the COC removed. A set of follicles were not microdissected to serve as controls. **B** Brightfield images of control follicles and unruptured sides displayed similar morphology with formation of a dark, round, smooth structure at 48 h post-ovulation under brightfield imaging. The ruptured side remained broken into small pieces that were lighter in color and less uniform. **C** Amount and distribution of cell death was similarly low in the control group and the unruptured sides. There were large pockets of dead cells present in the ruptured sides. **D** The amount of cell death was not significantly different between the control group and unruptured sides and was significantly elevated in the ruptured side. *n* = 15 follicles or follicle sides per group. **E** Hypertrophy of histologic sections was assessed using the Trainable Weka Segmentation plugin on FIJI to quantify the cytoplasmic-to-nuclear ratio. In the classified images, nuclei are colored in red, cytoplasm is colored in green, and background that is visible within the tissue section is classified as purple. **F** Throughout the 48 h period post-ovulation, the cytoplasmic-to-nuclear ratio increased in both the control group and the unruptured sides. The ruptured side exhibited a decrease in the cytoplasmic-to-nuclear ratio during the same period. *n* = 4–6 follicles or follicle sides per group. **G** An RNAscope assay demonstrated similar localization patterns of *Star* mRNA in the control group and the unruptured sides, which were distinct from the ruptured sides. **H** The amount of *Star* mRNA expression in the control and unruptured sides were significantly higher than the ruptured side. *n* = 4 follicles or follicle sides per group.

To determine whether there were fundamental differences in the ability of the distinct follicle sides to undergo luteinization in our ex vivo model, we cultured microdissected pieces independently for 48 h following ovulation induction and assessed key outputs of luteinization, including structural remodeling, cellular hypertrophy, and hormone production capacity (Fig. 2A). Non-microdissected follicles cultured for the same period served as controls. At 48 h post-ovulation, the control and unruptured sides formed uniform, spherical cellular aggregates that appeared morphologically similar by transmitted light microscopy (Fig. 2B). In contrast, the ruptured side formed irregularly shaped cellular aggregates as well as small clusters of cells rather than distinct structures (Fig. 2B). The majority of the cells in the control and unruptured side were viable after 48 h as assessed by a live-dead stain, whereas there was a greater percent of dead cells on the ruptured side (Fig. 2C, D, Supplementary Data 1). These findings demonstrate that the unruptured side of the follicle remains viable and functional post-ovulation, which would be expected of the side of the follicle that would be predicted to form the corpus luteum.

To determine whether there are functional differences between the follicle sides, we assessed cellular hypertrophy, a histologic hallmark of luteinization that occurs as the cytoplasm of luteal cells expands due to lipid production and steroidogenesis^93,94^. We compared the degree of cellular hypertrophy in distinct follicle regions by quantifying the cytoplasmic-to-nuclear ratio in histological sections (Fig. 2E, F, Supplementary Data 1). The cytoplasmic-to-nuclear ratio increases with increased hypertrophy, and this was observed similarly in the post-ovulatory period in both the control and unruptured sides (Fig. 2F)^95^. In contrast, the cytoplasmic-to-nuclear ratio decreased across the post-ovulatory period in the ruptured side (Fig. 2F). These findings demonstrated that the unruptured side of the follicle had a unique capacity to undergo cellular hypertrophy. To assess steroidogenic capacity of the unruptured and ruptured sides, we analyzed the expression of steroidogenic acute regulatory protein (*Star*) mRNA by in situ hybridization, which is highly expressed in CLs and regulates progesterone synthesis (Fig. 2G)^95^. There was higher *Star* expression in the control and unruptured follicles sides relative to the ruptured side at 48 h post-ovulation, which was consistent with corpus luteum function and the results of histologic analysis of hypertrophy (Fig. 2G, H, Supplementary Data 1). These results demonstrated that the unruptured side, when isolated after ovulation, remained functional and had a unique capacity, relative to the ruptured side, to undergo the structural and molecular changes associated with luteinization.

### The ruptured and unruptured sides of the follicle wall are molecularly distinct

**Fig. 3:**
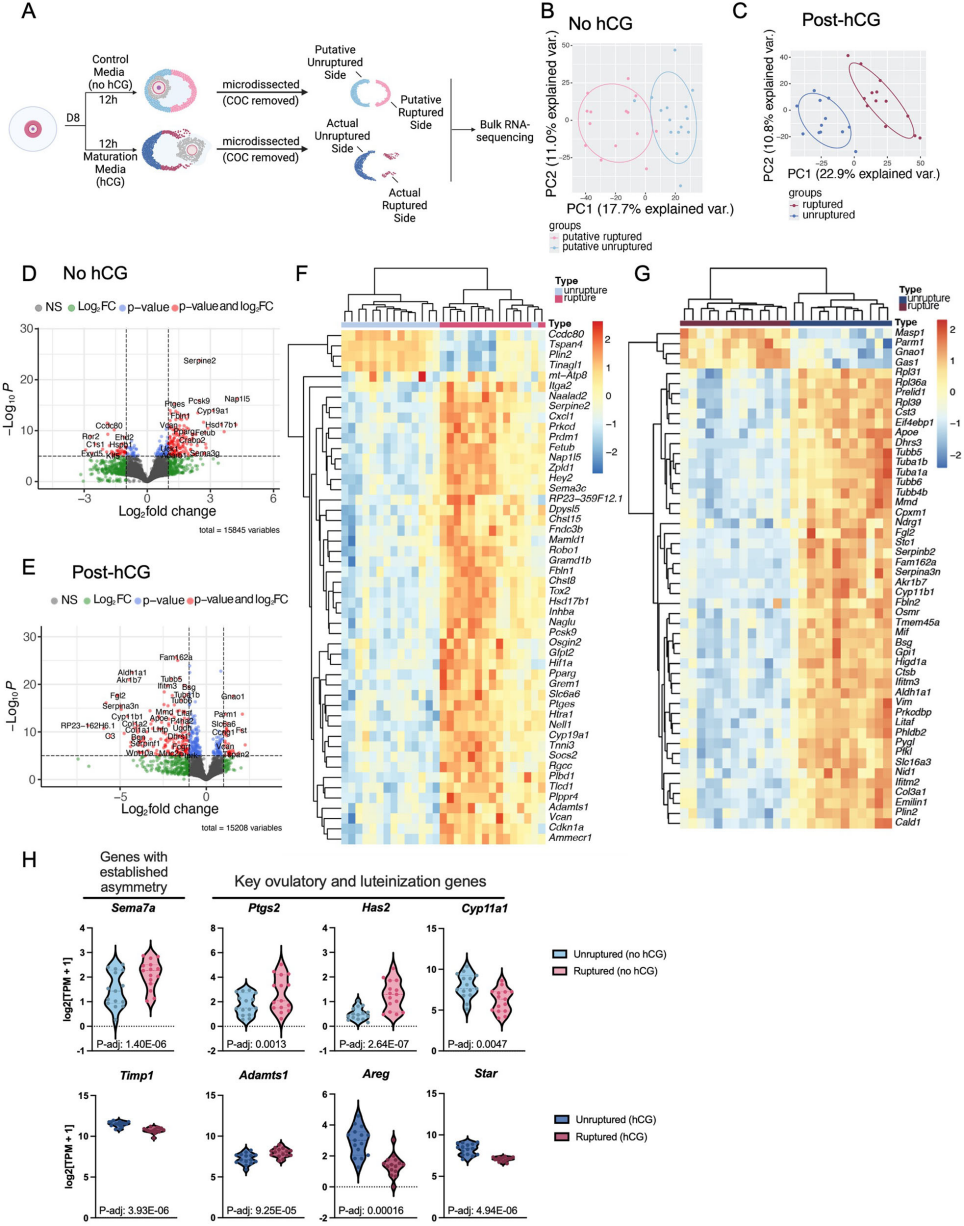
The unruptured and ruptured sides have distinct molecular signatures in both the no-hCG and post-hCG groups. **A** In our experimental model, we cultured follicles to the antral stage in alginate hydrogels. We then removed the follicles from the alginate and cultured them for 12 h in control media (no hCG) or maturation media enriched in hCG. After 12 h, we microdissected the follicles to separate the unruptured side from the ruptured side, and COCs were removed and discarded. Only the individual follicle sides were submitted for bulk RNA-sequencing. *n* = 15 follicle sides per group. **B** Follicle sides in the no-hCG group clustered by follicle side in a PCA plot. Ovals represent 95% confidence interval. Separation along PC1 in the no hCG comparison was driven by genes including collagen type III alpha 1 chain (*Col3a1*), serum amyloid A 3 (*Saa3*), inhibin subunit beta A (*Inhba*), and nucleosome assembly protein 1 like 5 (*Nap1l5*). **C** Follicle sides in the post-hCG group clustered by follicle side in a PCA plot. Ovals represent 95% confidence interval. In the post-hCG comparison, PC1 is driven by genes including aldo-keto reductase family 1 member B7 (*Akr1b7*), insulin like growth factor binding protein 3 (*Igfbp3*), insulin-like peptide 3 (*Insl3*), and aldehyde dehydrogenase 1 family member A1 (*Aldh1a1*). **D** There were 1,058 genes upregulated in the putative unruptured side (no hCG) and 1,041 genes upregulated in the putative ruptured side (no hCG). Red points on the volcano plot represent genes with a log2FC ≥ 1 and a -log10(P-adj) ≥ 5. Under these criteria, there were 128 genes upregulated on the putative unruptured side and 144 genes upregulated on the putative ruptured side. **E** There were 1,085 genes upregulated in the unruptured side (post-hCG) and 588 genes upregulated in the ruptured side (post-hCG). Red points on the volcano plot represent genes with a log2FC ≥ 1 and a -log10(P-adj) ≥ 5. Under these criteria, there were 358 genes upregulated on the putative unruptured side and 38 genes upregulated on the ruptured side. A heatmap of the top differentially expressed genes **F** between the putative unruptured and putative ruptured side and **G** between the unruptured and ruptured side demonstrates that follicle sides cluster together. **H** Our transcriptomic dataset recapitulates patterns of genes with known asymmetry from the literature (*Sema7a, Timp1*) and identifies several known ovulatory and luteinization-related genes as asymmetric during the periovulatory period (*Ptgs2, Has2, Cyp11a1, Adamts1, Areg, Star*).

We next wanted to profile the molecular signatures of each follicle side to better delineate the gene programs driving possible functional differences. To do this, we collected follicles at day 8 of culture and separated them into two groups: one that was exposed to hCG for 12 h to induce ovulation and another that was cultured but not exposed to hCG to serve as an unruptured control (Fig. 3A). Following the 12 h period, follicles in each group were microdissected to separate the unruptured side from the ruptured side (Fig. 3A). In the no-hCG group, these regions were referred to as the putative unruptured and ruptured sides, respectively, since they were microdissected based on predictive morphology (Fig. 1D–F). For both groups, the COCs were removed prior to performing bulk RNA-seq on individual follicle sides (Fig. 3A)^92,96,97^. High-quality sequencing data were obtained from these samples, with thousands of genes detected in each group, with a low fraction of reads mapping to ribosomal genes, and a comparable library size across each group (Supplementary Fig. 1). We also found that samples exposed to hCG were highly correlated, showing distinct clustering of ruptured and unruptured samples (Supplementary Fig. 1). We first performed principal component analysis (PCA) to identify global patterns in the data. The ruptured side and unruptured side samples separated along PC1 in both the no-hCG and post-hCG groups, demonstrating distinct transcriptomic features of the follicle sides (Fig. 3B, C).

In both the no-hCG and the post-hCG groups, there were several thousand differentially expressed genes (DEGs) with a false discovery rate (FDR) < 0.05 (Fig. 3D, E). In the no-hCG group, there were 1058 genes upregulated on the putative unruptured side (downregulated on the putative ruptured side) and 1041 genes upregulated on the putative ruptured side (downregulated on the putative unruptured side) (Fig. 3D, F). In the post-hCG group, there were 1085 genes upregulated on the unruptured side (downregulated on the ruptured side) and 588 genes upregulated on the ruptured side (downregulated on the unruptured side) (Fig. 3E, G). The top 10 upregulated genes in each group are shown in Table 1, and the complete list of all DEGs is available in Supplementary Data 2 and 3. Notably, our dataset recapitulates findings from previously published studies that report the asymmetric expression of specific genes during ovulation^12,31,32,54,55^. The gene Sema7a, a regulator of intercellular adhesion that is enriched at the site of rupture prior to ovulation in vivo, was enriched on the putative ruptured side (Fig. 3H)^55^. Tissue inhibitor of metalloproteinases 1 (*Timp1*), a protease inhibitor that localizes to the region opposite the side of rupture in vivo, was enriched on the unruptured side post-hCG (Fig. 3H)^31^.

**Table 1:** Top differentially expressed genes across comparisons between the putative ruptured side vs. putative unruptured side and the ruptured vs. unruptured side post-hCG

	Gene name	Description/Function	Log2 Fold Change	*P*-value (adjusted)
Top DEGs no hCG Putative ruptured (positive)	*Nap1l5*	Nucleosome assembly factor	4.275	5.33E-17
	*Inhba*	TGF-beta family	4.216	6.85E-12
	*Zpld1*	ECM constituent	3.654	1.61E-10
	*Hsd17b1*	Estrogen signaling dehydrogenase	3.519	6.57E-12
	*Paqr6*	Progesterone receptor	3.373	0.000242
	*Vwa5b1*	Von Willebrand factor extracellular protein	3.136	3.51E-05
	*Cyp19a1*	Estrogen biosynthesis enzyme	3.130	1.11E-14
	*Chst8*	Sulfotransferase	2.881	3.33E-11
	*Slc38a5*	Amino acid transporter	2.797	2.30E-07
	*Hsd11b2*	Cortisone signaling dehydrogenase	2.771	4.59E-08
Top DEGs no hCG Putative unruptured (negative)	*RP24-149G20.1*	Unknown	−3.160	0.0404
	*C1s2*	Serine protease	−2.928	6.19E-07
	*Hoxd10*	DNA-binding protein	−2.715	0.00429
	*Fxyd1*	Ion transporter	−2.712	5.72E-05
	*Ror2*	Tyrosine kinase	−2.690	1.33E-09
	*RP23-42H18.6*	Unknown	−2.677	0.0378
	*Stk32a*	Serine/threonine kinase	−2.649	8.45E-05
	*Fxyd5*	Ion transporter	−2.632	2.97E-06
	*F2rl3*	Thrombin-like protein	−2.615	0.0315
	*Ctss*	Peptidase	-2.610	0.00430
Top DEGs post-hCG Ruptured (positive)	*Adam22*	Metallopeptidase	2.254	5.34E-08
	*Gas1*	Cell cycle arrest protein	2.092	1.85E-14
	*Fst*	FSH release inhibitor	2.025	4.27E-11
	*Ear6*	Ribonuclease	2.013	0.00362
	*Cdh15*	Cadherin	1.896	2.60E-06
	*Tspan2*	Tetraspanin	1.720	4.86E-06
	*RP23-56C21.4*	Unknown	1.646	0.0110
	*Porcn*	Wnt signaling	1.575	1.71E-06
	*Kif2c*	Kinesin	1.561	0.00858
	*Gnao1*	Signal transduction	1.553	3.10E-18
Top DEGs post-hCG Unruptured (negative)	*Lama2*	Laminin	−7.435	4.94E-05
	*RP23-162H6.1*	Unknown	−6.863	4.40E-12
	*Ly6a*	Lymphocyte antigen	−6.816	0.00165
	*Insl3*	Insulin-like hormone	−5.951	1.79E-11
	*Serpinb2*	Endopeptidase inhibitor	−5.811	7.95E-17
	*C3*	Complement activation	−5.581	6.86E-10
	*Dhrs3*	Retinol metabolism	−5.170	1.78E-18
	*Fgl2*	Fibrinogen-like protein	−5.130	2.15E-18
	*Serpina3n*	Protease inhibitor	−4.972	3.16E-16
	*Col3a1*	Type III collagen	−4.937	1.83E-15

There was also differential expression of several key ovulatory genes, suggesting that their roles may be spatially-programmed during the process of follicle rupture (Fig. 3H). Prostaglandin-endoperoxide synthase 2 (*Ptgs2*), a known regulator of prostaglandin synthesis and inflammation that is essential for follicle rupture, was enriched on the putative ruptured side (Fig. 3H)^26,28,98^. Hyaluronan synthase 2 (*Has2*), a key regulator of cumulus cell expansion during ovulation, was also enriched on the putative ruptured side and may be important for cumulus migration towards the site of rupture (Fig. 3H)^22,99^. Cytochrome P450 cholesterol side-chain cleavage enzyme (*Cyp11a1*) is an essential regulator of progesterone synthesis during luteinization, was enriched in the putative unruptured side which may reflect early enrichment of progesterone production machinery (Fig. 3H)^100,101^. ADAM metallopeptidase with thrombospondin type 1 motif 1 (*Adamts1*), a metalloproteinase that is essential for normal follicle rupture, was enriched on the ruptured side post-hCG where it may play a role in the rupture-related remodeling (Fig. 3H)^27,102,103^. Amphiregulin, a regulator of cumulus expansion, progesterone production, and angiogenesis in the periovulatory period, was enriched on the unruptured side post-hCG which may be a driver of early luteinization in these cells (Fig. 3H)^104–107^. *Star*, another key driver of progesterone production, was also enriched in the unruptured side post-hCG (Fig. 3H)^21^. These results suggest that our assay recapitulated previously published findings on asymmetric pathways within ovulation and identified several key ovulatory genes that had asymmetric expression during the periovulatory period.

### Asymmetry of transcripts is conserved between ex vivo and in vivo ovulation

**Fig. 4:**
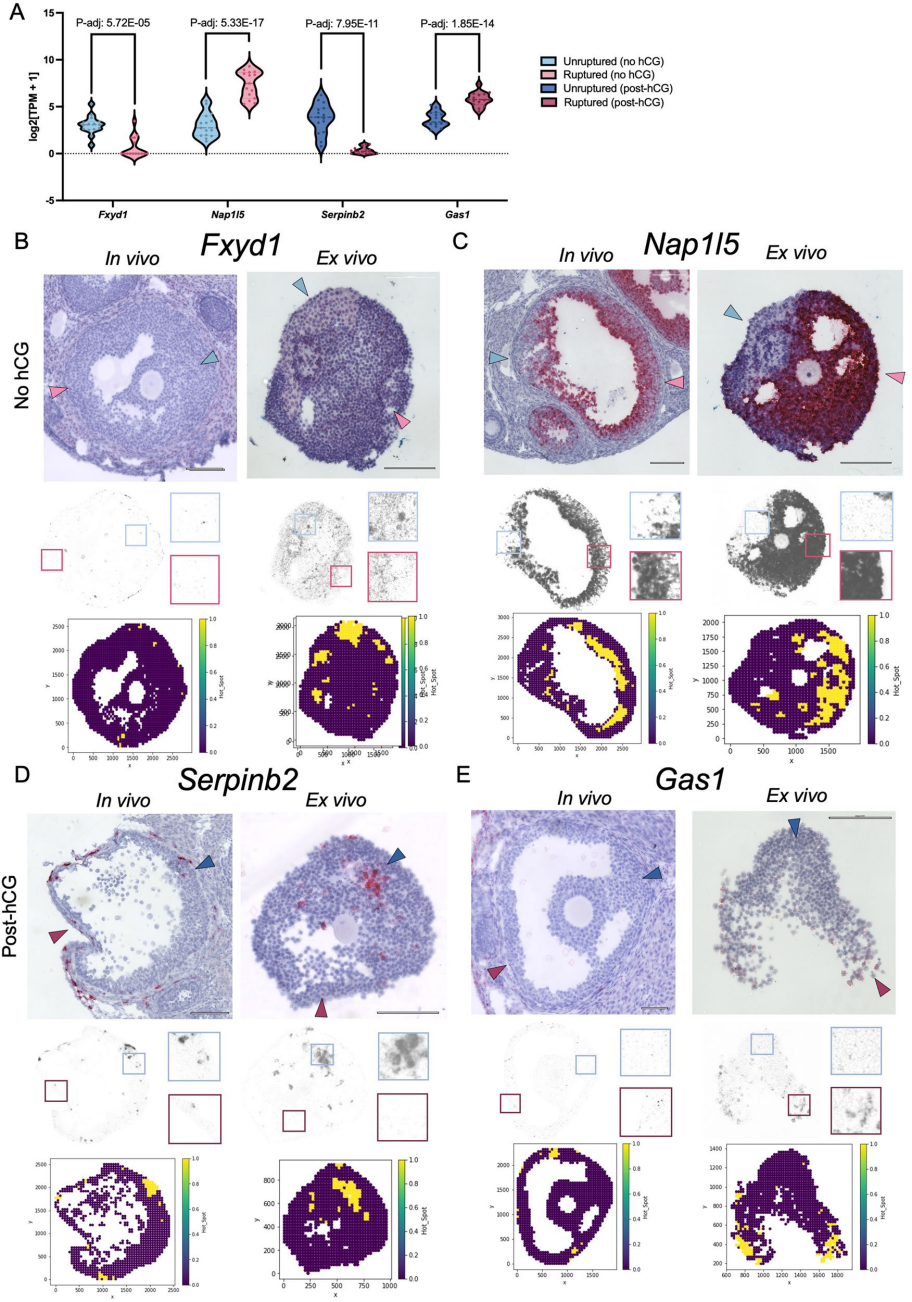
Asymmetric gene expression was validated using RNAscope in situ hybridization assays and was consistent between in vivo and ex vivo follicles. **A** Top differentially expressed genes (*Fxyd1, Nap1l5, Serpinb2, Gas1*) were selected from each comparison for validation. Violin plots are annotated with the adjusted p-value for each comparison. **B** Expression levels of *Fxyd1* were low in both in vivo and ex vivo follicles, but areas of higher signal were identified in the putative unruptured (PU) region of the follicle wall by both image deconvolution and Moran’s autocorrelation analysis. Boxed regions show a higher magnification section of the putative unruptured side (blue) and putative unruptured side (pink) in the color deconvoluted images, where dark spots represent mRNA signal. Yellow regions of Moran’s autocorrelation plots represent hot spots of locally elevated mRNA levels. **C** Asymmetric expression of *Nap1l5* demonstrated enrichment of mRNA in the putative ruptured side (PR) of the follicle wall, both through image deconvolution and Moran’s autocorrelation analysis. **D** *Serpinb2* expression was localized to around vasculature in vivo sections and identified hotspots in the unruptured region (U) of the ex vivo follicles. **E** *Gas1* expression was generally localized in the ruptured side (R) of the follicle wall in both in vivo and ex vivo sections, though the asymmetry is more dramatic in the ex vivo follicles. Representative images were chosen from 4–6 replicates per gene and per condition.

To validate the asymmetric expression of transcripts identified in our transcriptomic dataset and determine whether these patterns are relevant to in vivo ovulation, we performed RNA in situ hybridization to visualize several of the top differentially expressed transcripts within the follicle wall using histological samples from our ex vivo follicles and an in vivo time course of ovulation. We specifically validated the expression of FXYD domain containing ion transport regulator 1 (*Fxyd1*), nucleosome assembly protein 1 like 5 (*Nap1l5*), serpin family B member 2 (*Serpinb2*), and growth arrest specific 1 (*Gas1*), since they were among the top differentially expressed genes in the putative ruptured side, putative unruptured side, ruptured side (post-hCG), and unruptured side (post-hCG) respectively (Fig. 4A). To directly quantify asymmetry of these transcripts, we also used a computational strategy to identify hot spots of regionally elevated mRNA transcript levels using Moran’s autocorrelation analysis (Fig. 4B–E, Supplementary Fig. 2)^108^. In this analysis, hot spots are quantitatively identified to reveal spatial patterns of locally elevated mRNA expression relative to the surrounding area.

*Fxyd1* was highly expressed in the putative unruptured side without hCG exposure. Although the overall transcript level visible with the RNAscope in situ hybridization assay was low in the in vivo follicles, the identified hot spots are all generally localized on the putative unruptured side (light pink arrow) that is opposite of the thinning region of the ruptured side (light blue arrow) of the follicle wall (Fig. 4B). In the ex vivo sections, there were a larger number of mRNA transcripts detected that also had consistent asymmetry with higher expression on one side of the follicle wall (Fig. 4B). *Nap1l5* had highest expression on the putative ruptured side without hCG exposure. In our validation results, there was a clear spatial asymmetry of *Nap1l5* at the 0 h time point as shown by the yellow regions clustered on one side of the follicle wall (Fig. 4C). These clusters are localized to the region of wall thinning that is likely to form the site of rupture in both the in vivo and ex vivo representative follicles (Fig. 4C). *Serpinb2* had highest expression in the unruptured side 12 h post-hCG. At the 12 h time point, there were hot spots identified in the unruptured region both in vivo and ex vivo (Fig. 4D). Finally, *Gas1* had highest expression levels on the ruptured side at 12 h post-hCG. This pattern was present in the in vivo follicles post-hCG, with higher numbers of hot spots localized to one half of the follicle wall (Fig. 4E). In the ex vivo follicles, the asymmetry was more apparent and restricted to the ruptured region of the follicle wall. Asymmetry was also assessed in post-hCG follicles for *Fxyd1* and *Nap1l5* and in no hCG follicles for *Serpinb2* and *Gas1*, which also exhibited patterns consistent with the transcriptomic results (Supplementary Fig. 2). These results confirm our RNA-sequencing data and provide evidence that there are numerous genes within the follicle wall with asymmetric expression that may be relevant for follicle rupture and early luteinization.

### Transcriptomic results recapitulate known asymmetric processes and identify pathways that may drive follicle rupture and luteinization

**Fig. 5:**
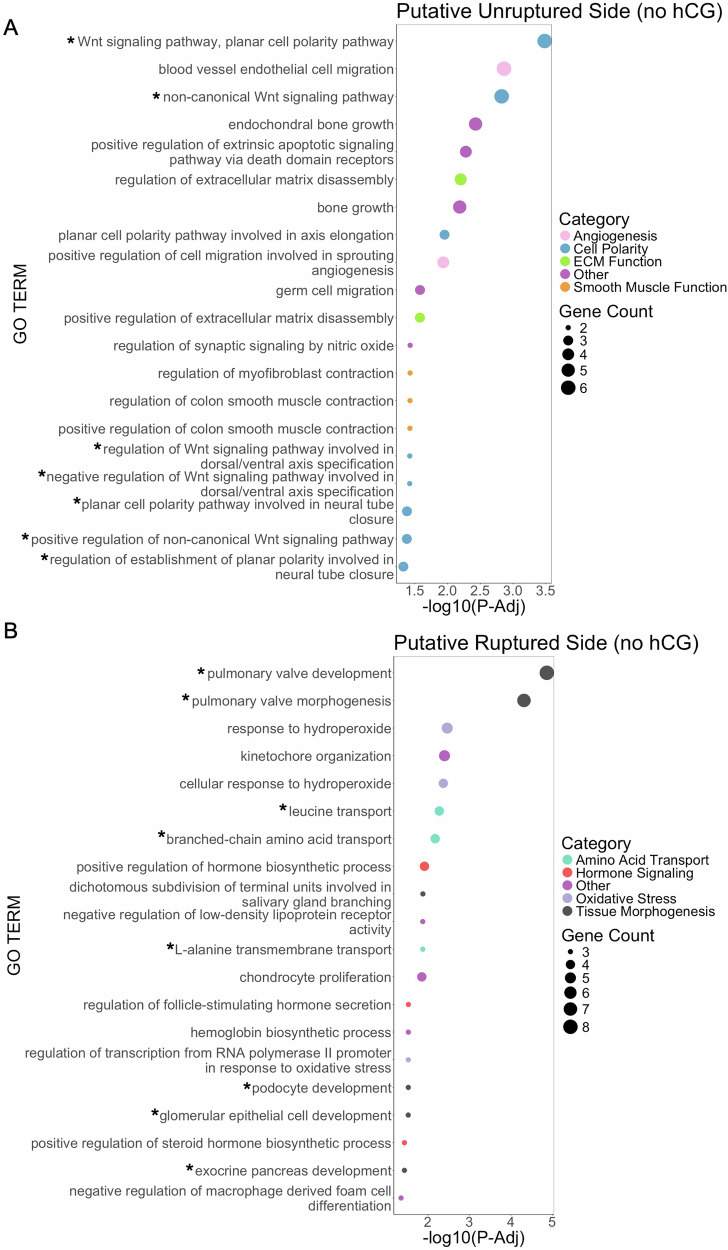
Pathway analysis identifies pathways that are differentially expressed between the putative unruptured and putative ruptured sides (no hCG). GO analysis for biological processes was completed on genes enriched in the (**A**) putative unruptured side and (**B**) putative ruptured side using Gene Ontology powered by PANTHER. The results were then manually categorized to identify trends in enriched pathways. Pathways marked by an asterisk are further described in the manuscript text and included in Table 2.

Overall, we found many genes that were differentially expressed between the ruptured and unruptured sides and between the putative ruptured and unruptured sides. To determine which pathways were being altered, we performed gene ontology analyses on these lists. In the putative unruptured side, there were enriched pathways related to angiogenesis and smooth muscle functions (Fig. 5A, Table 2). These pathways were driven by genes including junctional cadherin 5 associated gene (*Jcad)*, ras homolog family member J (*Rhoj*), and prostaglandin receptor 3 (*Ptger3*) (Table 2). Vasodilation, angiogenesis, and smooth muscle function all play a well characterized asymmetric role within the follicle wall^12,33–39,43–47,56–73^. Another set of highly enriched pathways were related to Wnt signaling and cell polarity. Although these pathways have not been previously described as asymmetric within the follicle wall, disruption of multiple Wnt family members is associated with subfertility and impaired corpus luteum formation^22,109–116^. Notably, deletion of *Wnt5a*, a gene that was enriched in the putative unruptured side in our dataset, leads to impaired fertility and progesterone production^109^.

**Table 2:** Genes driving biological processes identified from differentially expressed genes

Putative Ruptured vs. Putative Unruptured, no hCG				
	**Gene**	**Description/Function**	**Log2 FC**	**P-adj**
Angiogenesis	*Jcad*	Endothelial junction protein	−0.744	2.70E-05
	*Nrp1*	Cell migration regulator	−1.298	3.82E-06
	*Rhoj*	Endothelial adhesion and angiogenesis regulator	−1.547	3.58E-05
	*Hdac7*	Transcriptional regulator	−0.762	0.000487
	*Cxcl12*	Intercrine GPCR ligand	−1.114	5.65E-05
	*Robo4*	Regulator of cell adhesion and angiogenesis	−1.087	2.19E-05
	*Itgb1*	Integrin protein	−0.611	0.000745
	*Amotl1*	Tight junction component	−0.493	0.000641
	*Emp2*	Epithelial membrane protein	-0.575	1.92E-05
Smooth muscle function	*Ptger3*	Prostaglandin receptor	−1.469	0.000427
	*Kit*	Receptor tyrosine kinase	−1.694	1.24E-06
	*Pdpn*	Membrane glycoprotein	−1.304	0.000459
Amino Acid Transport	*Slc3a2*	L-type amino acid transporter	0.743	0.000958
	*Slc7a8*	L-type amino acid transporter	2.000	1.35E-05
	*Slc38a5*	Sodium-coupled amino acid transporter	2.797	2.30E-07
	*Slc7a6*	Basic amino acid transporter	0.449	7.36E-06
	*Slc6a15*	Neutral amino acid transporter	1.110	0.000161
Oxidative Stress	*Ddias*	DNA Damage response	1.066	0.000497
	*Prkcd*	Apoptotic regulator	1.084	1.11E-14
	*Trp53inp1*	Autophagy regulator	0.859	5.47E-07
	*Cd36*	Cell surface glycoprotein	1.698	5.20E-08
	*Hif1a*	Hypoxia inducible factor	1.245	4.97E-10
	*Epas1*	Oxygen responsive transcription factor	1.262	0.000252
	*Apoa4*	Apolipoprotein	2.235	5.04E-06
Tissue morphogenesis and Jag-Notch signaling	*Jag1*	Notch ligand	1.308	2.84E-06
	*Notch2*	Notch receptor	0.450	0.000584
				0.000459
**Ruptured vs. Unruptured, post-hCG**				
	**Gene**	**Description/Function**	**Log2 FC**	***P*-adj**
Response to copper	*Muc2*	Mucin protein	−2.173	2.96E-06
	*Mt1*	Metallothionein	−1.173	3.77E-08
	*Mt2*	Metallothionein	−0.997	7.09E-06
IL-11 signaling	*Jak1*	Protein tyrosine kinase	−0.369	0.000527
	*Il11*	Cytokine	−1.935	0.000984
	*Stat3*	STAT family member	−0.501	4.40E-09
Glycolytic processes	*Eno2*	Enolase enzyme	−1.373	7.61E-10
	*Tpi1*	Isomerase	−0.856	2.03E-13
	*Pkm*	Kinase	−0.667	7.19E-09
	*Pgk1*	Kinase	−0.892	6.51E-08
	*Gapdh*	Dehydrogenase	−0.318	0.000271
	*Galk1*	Kinase	−0.809	1.51E-12
	*Aldoa*	Aldolase	−0.394	1.75E-06
	*Hk1*	Kinase	−0.632	8.63E-06
	*Pfkl*	Kinase	−0.971	1.23E-16
	*Eno1*	Enolase enzyme	−0.641	2.65E-09
BMP signaling	*Fst*	Follistatin	2.025	4.27E-11
	*Heyl*	Transcription factor	1.165	0.000148
	*Comp*	Matrix protein	1.293	2.20E-06
	*Sfrp2*	Secreted frizzled receptor	1.064	1.63E-05
	*Inhbb*	Inhibin	1.396	4.31E-06
	*Notch2*	Notch receptor	0.403	0.000684
	*Runx2*	Transcription factor	0.752	7.44E-09
	*Bmper*	BMP binding protein	0.554	0.000152

In the putative ruptured side, the upregulated genes were enriched in processes related to amino acid transport, hormone signaling, oxidative stress, and tissue morphogenesis (Fig. 5B, Table 2). Specifically, the genes present in GO terms related to hormone signaling are involved in estradiol and follicle-stimulating hormone (FSH) signaling pathways. Oxidative stress related pathways were driven by genes including hypoxia inducible factor 1a (*Hif1a*), endothelial PAS domain protein 1 (*Epas1)*, and apoptotic regulators like protein kinase C delta (*Prkcd)* (Table 2). Oxidative stress and superoxide generation is known to play a role in driving follicle wall thinning and apoptosis during ovulation^117–119^. Amino acid transport related genes, including solute carrier family members *Slc3a2*, *Slc7a8*, and *Slc38a5*, were also highly enriched in the putative ruptured side and may play a role in amino acid transport to and from the maturing oocyte. Additionally, enriched pathways related to tissue morphogenesis are driven by signaling of jagged canonical notch ligand 1 (*Jag1*) and notch receptor 2 (*Notch2*). Jag-Notch signaling has a documented role in early follicle maturation, but any role that it may plan in ovulation has not been fully elucidated^120,121^. In both the putative unruptured and ruptured sides, we found enriched pathways that relate directly to known asymmetric processes during the periovulatory period as well as several pathways that have not been previously reported as asymmetric. Importantly, the known asymmetric processes from in vivo physiology are recapitulated in our ex vivo system even without exposure to hCG. This suggests that the pathways we have identified in this analysis represent ovulatory processes that are driven by follicle-intrinsic changes in gene expression that occur during this period independently of the ovulatory hormonal stimulus and the ovarian microenvironment.

**Fig. 6:**
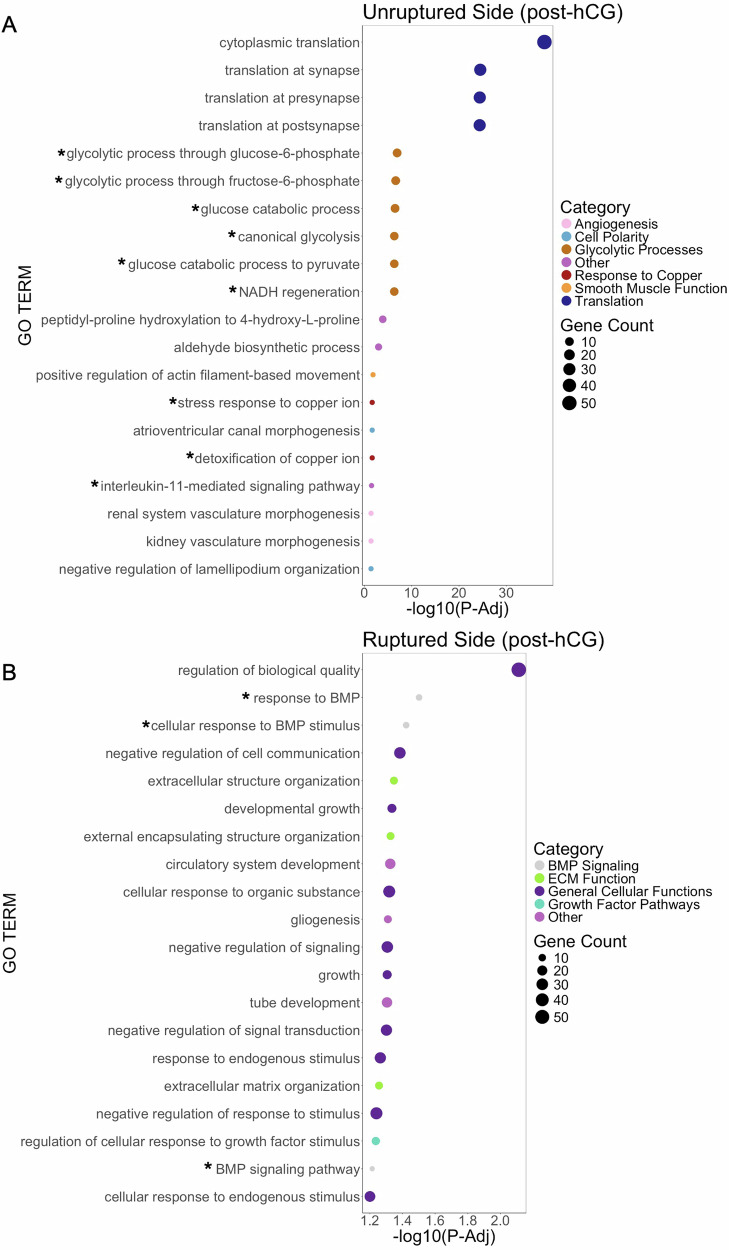
Pathway analysis identifies pathways that are differentially expressed between the unruptured and ruptured sides (post-hCG). GO analysis for biological processes was completed on genes enriched in the (**A**) unruptured side and (**B**) ruptured side using Gene Ontology powered by PANTHER. The results were then manually categorized to identify trends in enriched pathways. Pathways marked by an asterisk are further described in the manuscript text and included in Table 2.

In the post-hCG group, we similarly observed distinct gene expression patterns in the ruptured and unruptured sides (Fig. 6A). In the unruptured side post-hCG, the upregulated genes were enriched in angiogenesis, cell polarity, glycolytic processes, copper signaling, smooth muscle function, and translation (Fig. 6B, Table 2). Although several of these processes recapitulated known biology that differentiates the ruptured and unruptured sides, there are also several processes that have not been well characterized during ovulation or may be important for the spatio-temporal process of ovulation. Glycolysis-related pathways were highly enriched and have been recently shown to be essential drivers of early progesterone production in response to the ovulatory LH surge, which may be the basis of their asymmetric localization and enrichment on the unruptured side^122–124^. Other enriched processes include copper ion signaling pathways, driven by upregulation of genes like metallothionein 1 and 2, and IL-11 signaling which have not been previously reported as regulators of ovulation. In the ruptured side post-hCG, there were enriched processes related to bone morphogenic protein (BMP) signaling, ECM function, growth factor signaling, and general cellular functions (Fig. 6B). BMP signaling pathways, part of the TGF-beta super family, are known to play an important role in regulating ovulation^22,25,125–132^. Several other studies with knockout models of BMP pathway genes show a decreased ovulation rate and diminished ovarian function^125,127,128,132^. BMP receptor expression is high in oocytes, so it is possible that the observed asymmetry in our dataset reflects important communication between the ruptured side of the follicle and the cumulus oocyte complex^25^. The enriched genes in this dataset revealed a wealth of information about the spatially-distinct pathways that occur within the follicle wall in response to the hormonal trigger of ovulation.

### Transcriptomic analysis identifies distinct pathways that are enriched in the presence or absence of hCG on individual follicle sides

**Fig. 7:**
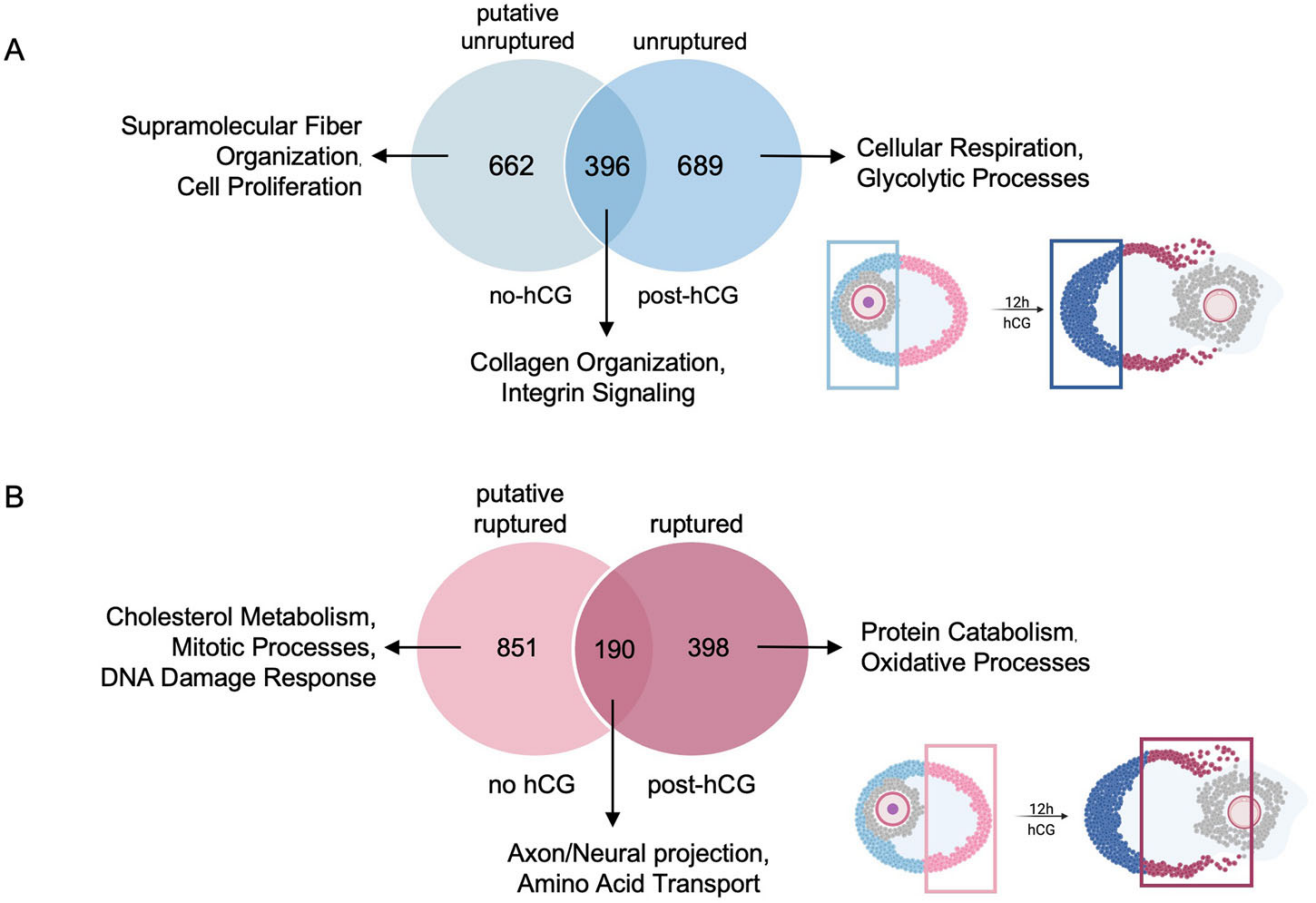
Pathway analysis on unique and common genes from DEG analysis on each follicle side reveals potentially spatiotemporally distinct processes during the periovulatory period. **A** When comparing the genes enriched in the putative unruptured side (relative to the putative ruptured side) and those enriched in the unruptured side (relative to the ruptured side), there were 622 genes unique to the putative ruptured side, 689 genes unique to the unruptured side, and 396 genes that overlapped between the two comparisons. Venn diagrams are annotated with top terms from pathway analysis on each resulting gene list. **B** When comparing the genes enriched in the putative ruptured side (relative to the putative unruptured side) to the genes enriched in the ruptured side (relative to the unruptured side), there were 851 genes unique to the putative unruptured side, 398 genes unique to the ruptured side, and 190 genes overlapping between the two comparisons. Venn diagrams are annotated with top terms from pathway analysis on each resulting gene list.

To further understand the molecular pathways that were enriched independently on the ruptured and unruptured side, we identified genes that were overlapping or unique on each side in the no hCG condition and the post-hCG condition. In total, there were 662 genes that were uniquely upregulated in the putative unruptured side (no hCG), 396 genes that were upregulated in both the putative unruptured side and actual unruptured side (post-hCG), and 689 genes that were uniquely upregulated in the unruptured side (Fig. 7A). The genes that were unique to the putative unruptured side were enriched in proliferative pathways and supramolecular fiber organization, which may suggest that the putative unruptured side may function to fortify the extracellular matrix of the corpus luteum and support cellular viability (Fig. 7A, Supplementary Table 1). The overlapping genes were enriched in pathways related to collagen organization and integrin signaling, suggesting that these fortification processes may continue after ovulation is induced hormonally (Fig. 7A, Supplementary Table 1). The genes that were unique to the unruptured side post-hCG are enriched in pathways related to cellular respiration and glycolytic processes, suggesting that the unruptured side is an important contributor to the glycolytic processes that support progesterone production in response to LH (Fig. 7A, Supplementary Table 1).

In addition, there were 851 genes that were uniquely upregulated in the putative ruptured side (no hCG), 190 genes that were upregulated in both the putative ruptured side and actual ruptured side (post-hCG), and 398 genes that were uniquely upregulated in the ruptured side (Fig. 7B, Supplementary Table 1). The pathways that were uniquely enriched in the putative ruptured side were related to cholesterol metabolism, mitotic processes, and DNA damage response. DNA damage response pathways may play an important role in responding to oxidative stress, while cholesterol metabolism pathways may be important for communication with the cumulus oocyte complex (Fig. 7B, Supplementary Table 1)^124^. Interestingly, top enriched pathways for genes that were upregulated in both the putative ruptured and actual ruptured sides were related to axonogenesis and neural projects, driven by genes including *Notch2*, plexin A1 (*Plxna1*), *Sema7a*, and EPH receptor A4 (*Epha4*) which have all been reported to be key pathways involved in communication between mural granulosa cells and cumulus cells during the periovulatory period (Fig. 7B, Supplementary Table 1)^133^. Finally, the genes unique to the ruptured side post-hCG were enriched in processes related to protein catabolism and oxidative processes, which is consistent with the functional phenotype of cell death we reported in this study (Fig. 7B, Supplementary Table 1). This analysis allowed us to identify pathways that may be both spatially and temporally regulated during the periovulatory period and further recapitulated known ovulatory biology and identifies previously unappreciated asymmetric pathways.

We also performed additional differential expression analysis was completed to compare the putative ruptured side (no-hCG) and the actual ruptured side (post-hCG) and to compare the putative unruptured side (no-hCG) and the actual unruptured side (post-hCG) (Supplementary Figs. 3-4, Supplementary Data 4-5). This analysis found several thousand genes on both the ruptured and unruptured sides that are differentially expressed in response to hCG (Supplementary Figs. 3-4). This comparison was important because it can reveal the genes and pathways that are specifically regulated by exposure to hCG in our system. On the unruptured side, the DEGs were enriched in pathways related to hormone stimulus and tissue morphogenesis in the no hCG putative unruptured side and transition to glycolytic and cholesterol production pathways post-hCG (Supplementary Fig. 3). The selective upregulation of both glycolytic and cholesterol-related pathways in response to hCG was consistent with known enrichment of progesterone production machinery in response to the LH surge^20,123^. On the ruptured side, the pathways enriched shifted from a variety of inflammation-related pathways in the no hCG putative ruptured side to glycolytic and metabolic pathways in the period post-hCG (Supplementary Fig. 4). This may suggest that the key inflammatory changes that are essential for follicle rupture, at least on a molecular level, may be encoded in the ruptured side prior to the LH surge^134^. This analysis revealed pathways that are active independently of hCG during the periovulatory period and those that are hCG-responsive and may contribute to rupture.

### Analysis of published single-cell transcriptomic datasets reveals cell type specificity of top enriched pathways

**Fig. 8:**
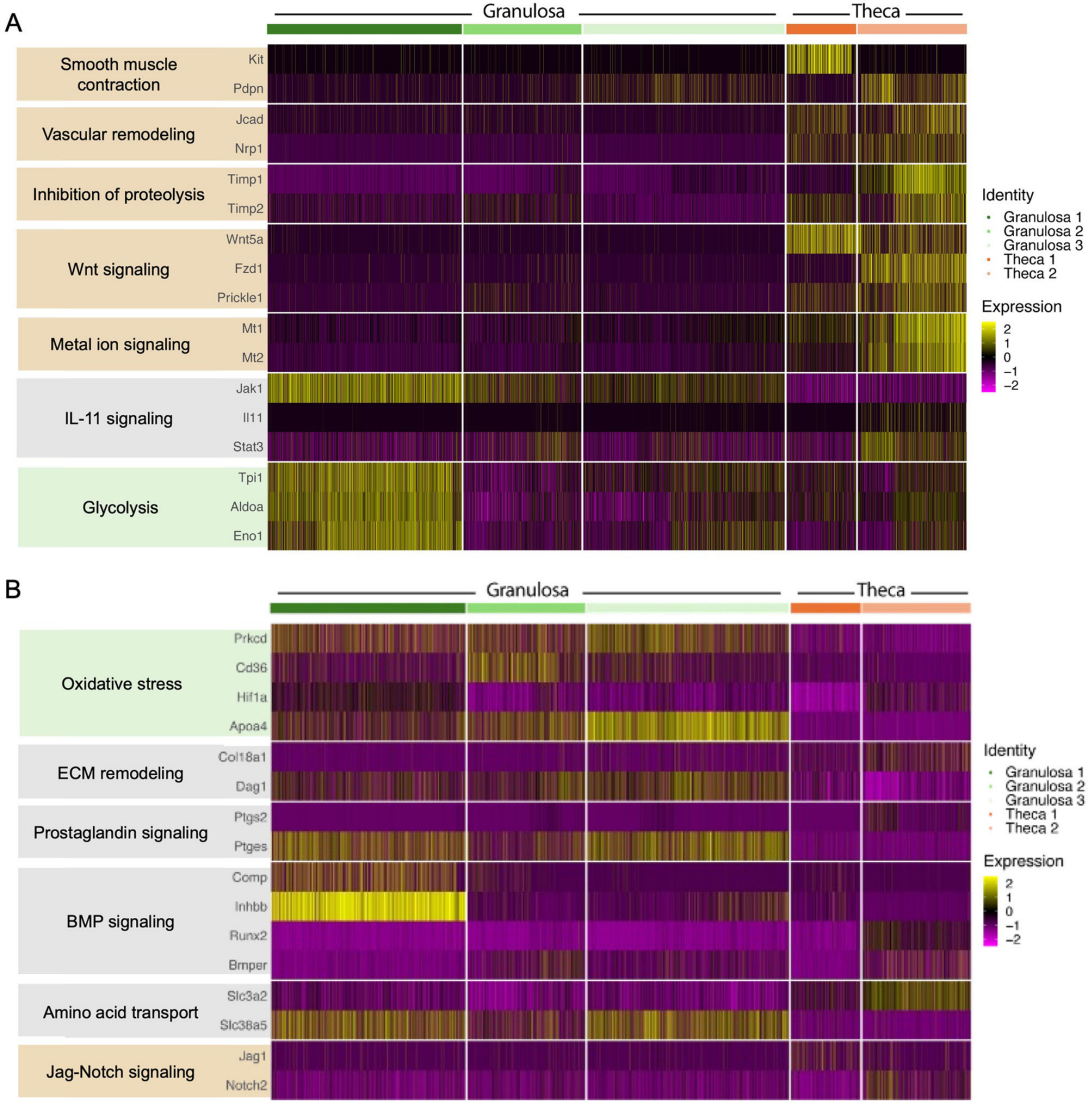
Analysis of published single-cell transcriptomic datasets reveals cell type specificity of top enriched pathways. Cell expression analysis derived from Huang et al., ^135^ identified cell type specificity for (**A**) genes enriched in the unruptured side and (**B**) genes enriched in the ruptured side.

To refine the cell types that drive individual asymmetric pathways within ovulatory follicles, we assessed gene expression patterns from published single-cell sequencing data^135^. In our system, the enzymatic isolation process breaks down the follicle basement membrane causing loss of much of the theca layer^136^. As follicles are grown in culture, they recover a theca layer that is hormonally active and grows throughout culture^136^. Since the COC was removed and discarded during microdissection, we expect that the sequenced samples consist primarily of granulosa and theca cells. To clarify whether key enriched pathways are driven by theca cells, granulosa cells, or both, we assessed expression of several DEGs in theca and granulosa clusters from a single cell sequencing dataset generated across the time course of ovulation (Fig. 8). Specifically, we analyzed expression of these genes in three identified granulosa cell clusters (granulosa 1, 2, and 3) and two identified theca cell clusters (theca 1 and 2).

On the unruptured side, we find that genes related to smooth muscle contraction and vascular remodeling are largely enriched in the theca layer, which is consistent with known ovarian biology (Fig. 8A). Genes related to inhibition of proteolysis, Wnt signaling, and metal ion signaling are also generally enriched in theca cells (Fig. 8A). IL-11 signaling exhibits mixed theca and granulosa cell expression, with *Jak1* enriched in the granulosa 1 cluster and *Stat3* and *IL11* both enriched in the theca 2 cluster (Fig. 8A). Genes related to glycolysis are enriched in the granulosa 1 cluster, consistent with the known central role of granulosa cells in metabolic function of follicles in response to ovulatory hormonal stimulation (Fig. 8A).

On the ruptured side, oxidative stress related genes were enriched across all three granulosa clusters (Fig. 8B). ECM remodeling had mixed expression between granulosa and theca cell types, suggesting that remodeling is occurring in both cell types (Fig. 8B). prostaglandin signaling, BMP signaling, and amino acid transport all had mixed expression patterns, which may indicate communication between theca and granulosa cells during the ovulatory period (Fig. 8B). Jag-Notch signaling, while lowly expressed, was largely restricted to the theca layer (Fig. 8B). This analysis further refined our pathway results to identify which cell types may participate in each of these key pathways.

## Discussion

The asymmetry of follicles undergoing ovulation in vivo has been described for decades and underlies release of a fertilization-competent oocyte and maintenance of the hormonal axis that supports establishment of pregnancy. The development of ex vivo models of ovulation that recapitulate the structural and molecular features of in vivo ovulation, including the asymmetry of follicle rupture, provides a new and important model system to study the follicle-inherent drivers of follicle rupture^79,91,92^. In this study, we demonstrated that the follicle wall consists of two distinct regions that drive follicle rupture and luteinization. By using an ex vivo model, we have demonstrated that this asymmetry is driven by processes intrinsic to the follicle and are independent of the ovarian surface epithelial cells, vasculature, immune infiltrates, and other stromal components.

Our study demonstrated that two distinct regions of the follicle wall, the ruptured side and unruptured side, had distinct functional phenotypes with respect to rupture and luteinization. The region of the follicle wall that ultimately ruptures largely underwent cell death and did not appear to luteinize. Cell death at the site of follicle rupture during the ovulatory period has been characterized previously in ovine models of ovulation^30,62–64,137–140^. It is thought that cell death at the site of rupture is essential for wall thinning leading up to rupture and clearing of the rupture site to allow for release of the COC^38,138^. This study is the first to demonstrate a spatially-distinct group of cells within the follicle wall that undergoes cell death using an ex vivo mouse model of ovulation. In contrast, the unruptured side of the follicle uniquely exhibited histologic and molecular hallmarks of luteinization, including cellular hypertrophy and increased *Star* expression. Thus, this region of the follicle wall had the capacity to form a functional corpus luteum.

Our transcriptomic dataset revealed a suite of genes, some shared and some unique, and pathways that may coordinate functions in a spatially-determined manner in the ruptured and unruptured sides of the follicle wall. We identified clear gene markers of the ruptured side, including *Nap1l5* and *Gas1*, and on the unruptured side, including *Fxyd1* and *Serpinb2*. These genes can serve as markers for these processes in vivo and can help guide future work to elucidate the exact functional programs of cells in these follicle regions. On the unruptured side, there were several top pathways that are consistent with known asymmetric processes during ovulation. Vascular signaling and smooth muscle contraction pathways were both enriched on the unruptured side. These pathways related directly to the known phenotypes of increased angiogenesis and contraction of smooth muscle within the unruptured side of the follicle wall^33–47,56–61,66–73,137,141,142^. Increased vascularization of the unruptured side and vasoconstriction of the ruptured side during ovulation has been documented across rodent, bovine, ovine, rabbit, and human studies and is essential for normal follicle rupture and luteinization^33,36–39,56–59^. Similarly, the enrichment of pathways related to smooth muscle function in our dataset is consistent with the known enrichment of smooth muscle contractility on the unruptured side of the follicle wall. This contractility has been demonstrated in hamster, non-human primate, and human studies of ovulation^34,35,42,43,47,72^. In mice, there is asymmetric localization of smooth muscle-like cells to the unruptured side of the follicle wall^35^. It is possible that these contractions play a role in mechanically pulling apart the follicle wall on the ruptured side and to assist in expulsion of the COC. The enrichment of angiogenic pathways on the unruptured side is particularly interesting given our ex vivo model in which the ovulatory follicle was isolated from the ovarian vasculature. The expression of these vascular genes in our ex vivo suggests that vascular regulation during ovulation may be driven by follicle-intrinsic factors despite lack of connection to the ovarian vasculature. Using single cell data, we found that the genes driving both vascular remodeling and smooth muscle related pathways were localized to theca cells. This is consistent with known in vivo localization of both peri-follicular vasculature and smooth muscle function during ovulation^12^. Finally, expression and localization of protease inhibitors such as tissue inhibitors of metalloproteinases (*Timp*) family members and serine protease inhibitor (*Serpin*) family members were enriched in the unruptured side^31,32,53,143^. This enrichment in the unruptured side is important for maintaining tissue and matrix integrity of the unruptured side as it begins to undergo luteinization^134^. In our dataset, we found enrichment of *Serpinb2*, *Timp1*, *Timp2*, and *Timp3* in the unruptured side after hCG exposure. These protease inhibitors may be essential regulators maintaining integrity of the unruptured side during the periovulatory period. Protease inhibitors may also drive the differences in morphology that we saw between follicle sides in the post-ovulatory period and the lack of cell death present on the unruptured side (Fig. 2B-D). Our analysis also demonstrated localization of key protease inhibitors to theca cell clusters. This is consistent with prior studies showing localization to the theca in vivo and may directly support luteal formation^31^.

This study also identified pathways that may be important asymmetric regulators of the unruptured side. Several top enriched pathways on the unruptured side were related to Wnt signaling and glycolytic pathways. Both pathways have been established as important for luteinization, but until this study they have not been shown to have direct asymmetric localization during the periovulatory period^109–116,123,124^. This transcriptomic result also supports our findings of luteinization capacity on the unruptured side. Our single cell analysis showed, as expected, that glycolytic processes were largely restricted to granulosa cell clusters^124,144,145^. Genes involved in Wnt signaling pathways were largely restricted to theca cell clusters, which has not previously been reported. We also found enrichment of pathways related to metallothionein signaling, classified under copper ion related processes in our gene ontology data. Metallothioneins are important contributors to sequestration of ROS and the detoxification of several heavy metals, including both copper and zinc. The importance of tight regulation of zinc levels for regulating fertility has been clearly demonstrated^146–152^. Less is known about the role that copper ions play in the ovary, though copper ions are known to induce granulosa cell death in ex vivo models^153,154^. Transgenic mice with deficient metallothionein 1a (*Mt1*) are infertile due to luteal insufficiency, suggesting that metallothioneins may play a crucial role in regulation of luteinization^155^. This is supported by a study that found elevated expression of *Mt1* mRNA in the luteinized granulosa cells of rat ovaries^155,156^. *Mt1* expression has also been shown to be localized to the theca layer during the ovulatory period, consistent with our single cell analysis^156^. Another top enriched pathway was related to IL-11 signaling. IL-11, a cytokine that has immune functions and acts upstream of Jak-Stat signaling pathways, has mostly been characterized in the context of the uterus^157^. Female mice with impaired IL-11 signaling are infertile due to decidualization defects^157–159^. Decidualization prepares the uterus for implantation of a blastocyst and is driven by the elevated progesterone levels that are produced by the corpus luteum that forms after ovulation^157^. Further studies show that, while circulating levels of IL-11 are low, there is production of IL-11 by ovarian cells that can be measured in follicular fluid and conditioned media from granulosa cells isolated from patients undergoing egg retrievals^160^. IL-11 also regulates ovarian steroidogenesis, stimulates progesterone production, and increases expression of *Star*^161^. Our cell type specificity analysis suggests that IL-11 signaling may be facilitated by communication between granulosa and theca cell layers based on expression of *Jak1* in granulosa clusters and expression of *Il11* and *Stat3* in theca clusters. Together with our findings, these studies suggest that the developing corpus luteum may be an important regulator of IL-11 signaling in advance of blastocyst implantation.

Similarly on the ruptured side, some of these pathways recapitulated what has been previously identified as asymmetric. For example, oxidative stress pathways were enriched in the putative ruptured side, which is consistent with the known role that superoxide generation plays in follicle rupture during ovulation. Treatment of mouse ovaries with antioxidants significantly suppresses ovulation, suggesting that generation of reactive oxygen species (ROS) is integral to oocyte release during ovulation^162^. A significant increase in oxidative damage in the region of rupture and the adjacent epithelium has been shown in an ex vivo sheep model^30,64^. Further, our analysis suggests that oxidative stress processes may be primarily localized to the granulosa cell layers and may play an important role in follicle wall thinning leading up to follicle rupture. Oxidative stress in the ruptured region may be an important driver of the cell death phenotype described in the literature and demonstrated in our ex vivo studies. In addition, *Sema7a* was enriched in the ruptured side, recapitulating recently published findings of asymmetry at the protein level^55^.

Beyond these pathways with a documented role in ovulation, there were several pathways enriched on the ruptured side whose role in follicle rupture has not been well-studied. The first of these pathways was related to amino acid transport via solute carriers. Amino acid transport is an essential process for oocyte maturation and provides nutritional support necessary for generation of fertilization competent oocytes^124,163–165^. In addition, cumulus cells support amino acid transport to the oocyte through solute carrier channels^124,163,165^. There is some evidence that pre-ovulatory mural granulosa cells also facilitate amino acid transport to the oocyte, as transport of several key amino acids was highest in intact follicles compared to oocytes and granulosa cells alone, and infusion of branched-chain amino acids during the late luteal phase leads to increased ovulation rates in ewes^164,166,167^. Our analysis suggests that both theca and granulosa cells may participate in amino acid transport, with specific solute carrier channels localized to each cell type. We also identified Jag-Notch signaling as an enriched pathway on the ruptured side. Jag-Notch signaling in the ovary has largely been characterized in the context of primordial follicle activation. Suppression of notch signaling in neonatal mouse ovaries leads to reduced primordial follicle assembly, suggesting an integral role of Jag-Notch signaling in breakdown of germ cell nests and formation of the ovarian reserve^120,121,168–171^. Importantly, both *Notch2* and *Jag1* are expressed in granulosa cells of antral stage follicles^172,173^. In our cell type analysis, we found low expression of *Jag1* and *Notch2* across both theca clusters. Existing studies that explore the function of Jag-Notch signaling in antral-stage follicles demonstrate a role of the signaling pathway in regulating proliferation, apoptosis, and steroidogenesis^120,170,174–176^. Interestingly, a knockout mouse model of the lunatic fringe (*Lfng*) gene, an upstream regulator of Jag-Notch signaling, shows impaired follicle rupture during ovulation and increased rates of trapped oocytes within corpora lutea^177^. These studies in combination with our findings may indicate a role of asymmetric amino acid transport and Jag-Notch signaling as a driver of follicle rupture.

Although it is not yet known what drives the asymmetry within antral follicles, this asymmetry is essential for formation of a follicle rupture site. When the follicle is situated within the ovarian microenvironment, there are likely biomechanical and molecular signals that drive the asymmetry in a particular direction to facilitate follicle rupture adjacent to the ovarian surface as a prerequisite for COC release. When the follicle is removed from this environment, we have demonstrated that asymmetry is maintained and similarly dictates rupture of the follicle. Our findings demonstrate that there are follicle-intrinsic mechanisms that promote asymmetric antrum formation, and this does not appear to be an artifact of the culture conditions as it is observed in non-encapsulated ex vivo systems as well^178^. Nevertheless, extrinsic factors from the ovarian microenvironment also play a clear role in driving this asymmetry in vivo. The ovarian microenvironment may play a particularly important role in establishing the directionality of the asymmetry and to ensure release of the egg at the correct time and place to provide for optimal fertilization potential through contributions of the stroma, vasculature, and epithelial components^12^. Novel ex vivo follicle culture strategies that incorporate features of the ovarian microenvironment on a structural or molecular level will be essential tools to further elucidate the factors required for optimal follicular rupture and ovulation^178,179^. In addition, though our study used a machine learning guided microdissection technique to identify follicle sides, there are limitations to our approach. Ovulatory follicles are complex structures that may have a gradient of molecular and morphologic changes that cannot be captured by microdissection of the follicle wall grossly into two discrete sections. Future studies incorporating spatial transcriptomics or whole-ovary immunofluorescence with tissue clearing may provide a higher degree of spatial resolution than was possible with our technique^135,180–182^. Despite this limitation, we still identified thousands of differentially expressed genes and pathways of biological interest demonstrating that follicle asymmetry during ex vivo ovulation is a robust phenotype.

**Fig. 9:**
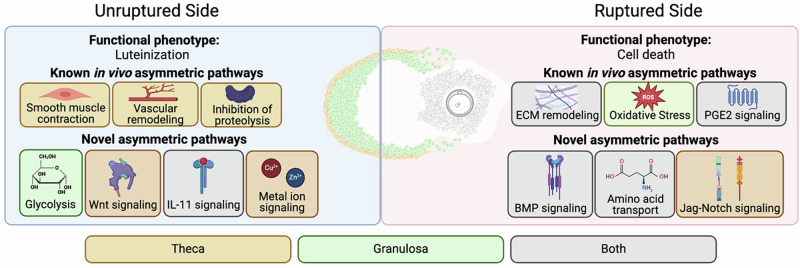
Summary of key findings. Distinct regions of the ex vivo follicle wall exhibit functional and molecular differences that both recapitulate known ovulation biology and represent novel pathways that may participate in asymmetric functions during the periovulatory period. These pathways, identified through gene ontology, represent areas of potential biological significance for the processes of ovulation and luteinization. Further analysis identified cell-type specificity of each pathway. Some genes underlying key pathways were enriched primarily in granulosa cells (green), theca cells (orange), or a combination of both cell types (gray). The cumulus-oocyte-complex, which was excluded from our analysis, is shown in black and white.

In summary, our study demonstrated that there are a series of core ovulatory mechanisms which are autonomous to the follicle itself and can occur even in the absence of the ovarian context. Secondly, we showed that regions of the ovulatory follicle perform distinct actions and have unique expression profiles during the periovulatory period. Specifically, the ruptured side of the follicle wall undergoes remodeling, oxidative stress, and eventually cell death (Fig. 9). Concurrently, the unruptured side is fortified with proteolysis inhibitors, angiogenesis, and smooth muscle activity prior to luteinization (Fig. 9). We identified pathways that may play an important role in the asymmetric processes underlying ovulation including Wnt signaling, glycolytic processes, metallothionein function, IL-11 signaling, amino acid transport, Jag-Notch signaling, and BMP signaling (Fig. 9). Finally, we harnessed a published single cell dataset to identify cell-type specificity of each of these asymmetric pathways to further elucidate their potential function in the periovulatory period (Fig. 9). Beyond the identification of specific previously unexplored pathways, the transcriptomic dataset generated in this study may serve as an important resource for advancing human health and fertility. First, this dataset could be used to identify potential non-hormonal contraceptive targets. Pathways that function primarily on the ruptured side may be targetable to block follicle rupture without preventing luteinization. Several genes enriched in the ruptured side in our study, including *Insr*, *Adamts1*, and *Ptgs2*, have knockout models which display the phenotype of a trapped oocyte within a developed corpus luteum^23,27,98,183^. Although these genes may not directly be ideal contraceptive targets, they may be part of pathways that could be targetable. Beyond contraceptive development, identifying molecular markers of follicle rupture may be critical for identifying mechanisms of idiopathic anovulatory infertility. Genes identified in this dataset may be impaired in women with unexplained infertility and may identify future clinical interventions to improve assisted reproductive technology outcomes in these patients.

## Methods

### Animals

CD-1 adult female mice were purchased with 5-day-old female pups (Envigo, Indianapolis, IN). Pups were housed in polypropylene cages in the animal facilities of Northwestern University for a minimum of 7 days with the adult breeder female after arrival to ensure acclimation prior to use. Mice were kept under a temperature-, humidity-, and light-controlled barrier facilities (14-hour light/10-hour dark cycles) and were provided with food and water *ad libitum*. All animal procedures were approved by the Northwestern University Institutional Animal Care and Use Committees (IACUC). We have complied with all relevant ethical regulations for animal use.

### Follicle isolation and eIVFG

Mouse secondary follicles were isolated from the ovaries of 14-16 day-old CD-1 female mice enzymatically using L15 media (Thermo Fisher Scientific, Waltham, MA) with 1% penicillin-streptomycin (Thermo Fisher Scientific), 25 μg/ml liberase (Sigma-Aldrich, St. Louis, MO), and 200 μg/ml DNase I (Qiagen, Hilden, Germany)^75–79,81–85,87,91,92^. After 20 minutes of exposure, the enzymatic solution was quenched with 10% fetal bovine serum (FBS; Peak Serum Inc., Wellington, CO) and ovary pieces were manually dispersed using a P1000 pipette to release follicles. Follicles were classified and selected based on morphology and size (150–180 μm diameter). Follicles with intact morphology were encapsulated ina 0.5% (w/v) alginate hydrogel (Sigma-Aldrich) for the preservation of the 3D structure of follicles throughout folliculogenesis. Follicles were individually placed within 5 μl alginate hydrogel beads that were then submerged in 50 mM CaCl2 (Thermo Fisher Scientific) and 140 mM NaCl (Thermo Fisher Scientific) for 2 min to allow for crosslinking of the alginate polymers. Individual encapsulated multi-layer secondary follicles within alginate beads were cultured in 96-well plates. Each well contained one alginate bead and 100 μl growth media, which consists of 50% αMEM Glutamax (Thermo Fisher Scientific) and 50% F-12 Glutamax (Thermo Fisher Scientific) supplemented with 3 mg/ml bovine serum albumin (BSA; Sigma-Aldrich), 10 mIU/ml recombinant follicle stimulating hormone (rFSH; Gonal-F, Merck & Co., Rahway, NJ), 1 mg/ml bovine fetuin (Sigma-Aldrich), and 5 μg/ml insulin-transferrin-selenium (ITS, Sigma-Aldrich). Follicles were cultured at 37 °C for 8 days in a humidified environment of 5% CO2 in air with half of the growth media replaced daily. As follicles are cultured individually in our system and were therefore not exposed to paracrine factors from other nearby follicles, we removed the follicle selection and atresia that occurs in vivo^91^. We completed follicle selection during the follicle isolation process to only select the most optimal follicles for culture, which provided artificial follicle selection and allowed for a yield of more than 80% of follicles that survived and matured to the antral stage^91^.

### Ex vivo ovulation and luteinization assay

After day 8 in culture, antral stage follicles (diameter ≥ 350 μm) were removed from alginate beads by incubating in L15 media containing 1% FBS (Peak Serum Inc.) and 10 IU/ml alginate lyase from flavobacterium multivorum (Sigma-Aldrich) at 37 °C for 20 min. Follicles were then washed in L15 media (Thermo Fisher Scientific) containing 1% FBS (Peak Serum Inc.) and transferred to 100 μl of maturation media containing 50% αMEM Glutamax (Thermo Fisher Scientific) and 50% F-12 Glutamax (Thermo Fisher Scientific) supplemented with 10%FBS (Peak Serum Inc.), 1.5 IU/ml human chorionic gonadotropin (hCG; Sigma-Aldrich), 10 ng/ml epidermal growth factor (EGF; BD Biosciences), and 10 mIU/ml rFSH (Merck & Co.) or control media which contained the same components except for hCG in individual wells of a 96-well plate. Follicles were incubated in maturation media for 12 h in a humidified environment of 5% CO2 in air to allow for ovulation and follicle rupture. For luteinization experiments, after 12 h of exposure to maturation media to induce follicle rupture, conditioned media was collected and stored at −20 °C for ELISA and follicles were washed in L15 media (Thermo Fisher Scientific) supplemented with 1% FBS (Peak Serum Inc.). Post-ovulatory follicles were microdissected to remove the COC and separate the ruptured side from the unruptured side. The follicle sides were placed in 100 μl of media containing 50% αMEM Glutamax (Thermo Fisher Scientific) and 50% F-12 Glutamax (Thermo Fisher Scientific) supplemented with 10% FBS (Peak Serum Inc.) and incubated for 48 h in a humidified environment of 5% CO2 in air to allow for ovulation and follicle rupture. Conditioned media was collected after 48 h and stored at −20 °C for ELISA. Tissue pieces were then either used for ATP quantification or fixed in 3.8% PFA with 0.1% Triton X at 37 °C for 1 h for histology or in situ RNA hybridization.

### Machine learning imaging analysis of asymmetry

Manual image annotation was performed on 92 day 8 follicles removed from alginate and their corresponding image after ovulation induction using the v7 data labelling system, with the labels ‘unruptured’ and ‘ruptured’ for the distinct follicle regions. The training subset of data was applied to a deep-learning based object detection model within an autonomous training routine. The object detection model used was a Mask R-CNN model with a ResNet-50-FPN backbone. Model performance was validated regularly throughout the training process on the unseen evaluation set. Average precision and average recall were used as evaluation metrics. Data augmentation techniques (including albumentations ‘CoarseDropout’ and ‘ShiftScaleRotate’) were applied during training to prevent overfitting on the small amount of data, therefore providing a robust model with the ability to generalize to future unseen datasets. A method called Class Activation Map (CAM) was used to interrogate the object detection model for the morphological features that were most important for the prediction of the ruptured and unruptured follicle regions. These activation maps are a useful validation method to ensure the most important features to the model are biologically relevant, rather than the generated predictions being based on inconsequential details in an image.

### Follicle microdissection

Antral stage follicles were microdissected using insulin gauge needles (BD Biosciences, Franklin Lakes, NJ) in L15 media (Thermo Fisher Scientific) supplemented with 1% FBS (Peak Serum Inc.) to separate the putative ruptured side from the putative unruptured side in the control follicles (incubated for 12 h in maturation media without hCG) or the actual ruptured side from the actual unruptured side in follicles after 12 h in maturation media. In both groups, the COC was removed after microdissection. The site of microdissection in control follicles was identified based on the swelling of the antral cavity, a morphologic hallmark of the site of rupture, and guidance from the machine learning-based imaging analysis.

### Live-dead assay

Cell death and survival was quantified using calcein-AM and ethidium homodimer to stain for live and dead cells respectively. Samples were incubated with 4 µM calcein-AM and 8 µM ethidium homodimer for 30 min at 37 °C and were protected from light. Following incubation, samples were washed 3x with phosphate buffered saline (PBS) and plated in a drop of PBS on a glass bottom dish. Samples were then imaged with a Leica TCS Sp5 confocal system using 40× objectives to take Z-stack images throughout each sample (Leica Microsystems). Ratio of live to dead cells was quantified using ImageJ by calculating the area of the red eithidium homodimer positive area and dividing it by the area of the green calcein-AM area on a max intensity projection of the z-stack.

### Mouse ovary sample collection

4-6 week-old CD-1 female mice were treated with 5 IU pregnant mare serum gonadotropin (PMSG; Calbiochem, San Diego, CA) via intraperitoneal injection to stimulate follicle maturation. After 46 h, the same mice were injected with 5 IU hCG (Sigma-Aldrich) via intraperitoneal injection to induce ovulation. Mice were sacrificed to collect ovaries at 0 and 12 h post-hCG injection. Ovaries were fixed in Modified Davidson’s solution (Electron Microscopy Services, Hatfield, PA) at room temperature for 2–4 h with agitation. Tissues were then kept in fixative at 4 °C overnight with agitation. After, samples were washed three times in 70% ethanol and kept at 4 °C prior to processing for histology.

### Preparation of fixed tissue for histology

Fixed follicles, microdissected follicle sides, samples from luteinization assays, and ovaries were processed using an automated tissue processor (Leica Biosystems, Buffalo Grove, IL) per standard processing protocols. Tissues were then embedded in paraffin wax and serially sectioned at 5  μm intervals and placed on slides. Some sections were selected for staining with hematoxylin and eosin (H&E, Thermo Fisher Scientific) for histologic evaluation. Nuclear to cytoplasmic ratio in samples from the luteinization assay was calculated in ImageJ using the plugin Trainable Weka Segmentation to identify nuclear area and cytoplasmic area^184^.

### Bulk RNA-seq

Libraries for RNA-seq were generated from individual microdissected follicle sides for a single cell workflow with fewer amplification cycles. Smart-Seq2 cDNA synthesis was performed on RNA isolated from follicle sides with Maxima H Minus reverse transcriptase (Thermo Fisher). Whole transcriptome amplified (WTA) product was purified with AMPure XP beads (Beckman Coulter), quantified with Quanit-iT PicoGreen dsDNA Assay (Thermo Fisher), normalized, and used for preparing paired-end sequencing libraries with Nextera XT (Illumina, #FC-131) using the manufacturer’s instructions. Libraries were pooled equally before sequencing on a NextSeq500/550 (Illumina) using a 75 cycle v2 sequencing kit.

### Analysis of transcriptomic data

After sequencing, BCL files were converted to FASTQs that were merged and demultiplexed. FASTQs were assessed for size to ensure that sequencing depth was comparable across all samples. They were then mapped to the GRCM38 genome using HISAT2 and quality control metrix were extracted using publicly available workflows on Terra (https://portal.firecloud.org/?return=terra#methods/GP-TAG/SS2_scRNA_pipeline/32). Following alignment, the count data was batch-corrected with ComBat-seq^185^. Batch-corrected and converted Transcripts Per Million matrices were then used on all subsequent analyses. Differential expression analysis was completed using the DESeq2 package in R^186^.

### RNAscope in situ hybridization assay

To validate the RNA-seq results and assess the spatiotemporal expression pattern of top DEGs, in situ RNA hybridization was performed on histologic sections from cultured follicles and ovaries using the RNAscope 2.5 HD Detection Kit RED and HybEZ Hybridization System (Advanced Cell Diagnostics, Inc., Newark, CA). The protocol was optimized according to the manufacturer’s instructions. Tissue sections were treated with heat, hydrogen peroxide, and protease treatment before treatment with the target gene probes and amplification system. The probes were then labeled with a chromogenic red dye after which the tissue was stained with hematoxylin and eosin and mounted for imaging with an EVOS cell imaging system (Thermo Fisher Scientific).

### RNAscope quantification for asymmetry

Validated genes were selected based on the top differentially expressed gene in each of our four comparisons with a TPM value of ≥ 3 in the region of interest, excluding mitochondrial genes and genes without functional RNAscope probes. To assess spatial asymmetry of top DEGs within the antral follicle wall, regions of interest from tissue sections treated with the RNAscope in situ hybridization assay were computationally processed for Moran’s Local Autocorrelation analysis to identify regions of relatively elevated mRNA levels. Antral follicles from tissue sections were isolated in ImageJ and processed using the ImageJ plugin Trainable Weka Segmentation to identify probe and cell area according to protocols published by the RNAscope manufacturer. After segmentation and pseudocoloring, a grid of square regions of interest the size of an average cell in the follicle wall were added in ImageJ. The image was then split into thresholds to separate the cell area from the probe area. Measurements of cell area and probe count per region of interest were then taken along with the coordinates of the center of the region. These data points were used in the python package Python Spatial Analysis Library (PySAL) to compute Moran’s local autocorrelation statistics and plot regions of elevated local Moran’s statistics^108^.

### Analysis of single-cell transcriptomic datasets for cell type specificity

Cell Ranger was used for demultiplexing after acquiring the raw sequencing data as well as the subsequential alignment^187^. Seurat 4.2 was used in R 4.2.2 was used for initial quality filtering which removed cells with greater than 20% mitochondrial gene expression or less than 3000 expression counts^188–192^. The dataset then went through scaling, normalization, dimension reduction, doublet removal, and clustering, which is how granulosa and theca cell clusters across the time course of ovulation are subsetted. The heatmap shows the expression of those genes in this single-cell dataset.

### Statistics and reproducibility

Statistical analysis was performed using GraphPad Prism 9.0 (GraphPad Software Inc., San Diego, CA, USA). Data in figures are represented as the mean ± standard deviation. When comparing nuclear to cytoplasmic ratio, StAR mRNA expression, and live/dead cell ratio, a two-way ANOVA with Tukey correction for multiple comparisons was used to analyze the statistical significance between groups. A *p*-value < 0.05 was considered statistically significant. Sample are noted in the figure legend of each dataset and raw data for generated graphs is included in Supplementary Data 1.

### Reporting summary

Further information on research design is available in the Nature Portfolio Reporting Summary linked to this article.

## Supplementary information

Review file

Supplementary Information

Description of Additional Supplementary Files

Supplementary Data 1

Supplementary Data 2

Supplementary Data 3

Supplementary Data 4

Supplementary Data 5

Reporting Summary

## Data Availability

Associated RNA-seq data can be found at the Gene Expression Omnibus (GSE266449). All other data are available from the corresponding author on reasonable request.

## Code availability

All coding used to analyze RNA-sequencing data and complete Moran’s Autocorrelation analysis was used from publicly available resources.
