# Supplementary material for: Follicle-intrinsic and spatially distinct molecular programs drive follicle rupture and luteinization during ex vivo mammalian ovulation
Source: Commun Biol. 2024 Oct 23;7:1374. doi: 10.1038/s42003-024-07074-9 (PMC11500180; doi:10.1038/s42003-024-07074-9)
Supplement: Supplementary file 2 — Supplementary Information [file 42003_2024_7074_MOESM2_ESM.pdf]

Supplementary Figures

Supplementary Figure 1. All sequenced samples were of high quality.

All sequenced samples had similar numbers of A) genes detected, B) % ribosomal content, and C) estimated library size. D) Samples cluster together based on media conditions (hCG and no hCG).

Supplementary Figure 2. RNAscope *in situ* hybridization assay demonstrates consistent expression patterns with sequencing results in non-enriched sample groups.

A) Expression levels of top enriched genes within the other media condition. B-D) All four genes exhibited lower expression levels in the media condition that was not shown in Figure 3. The patterns for genes with detectable expression (*Fxyd1* and *Nap1l5*) were consistent with the transcriptomic results. Representative images were chosen from 4-6 replicates per gene and per condition.

Supplementary Figure 3. Additional comparisons between putative unruptured side and unruptured side (post-hCG).

A) 3,330 genes were enriched in the putative unruptured side and 2,994 genes were enriched in the unruptured side post-hCG. B) Samples clustered together by media condition. GO analysis for biological processes was completed on genes enriched in the C) putative unruptured side and D) unruptured side using Gene Ontology powered by PANTHER.

Supplementary Figure 4. Additional comparisons between putative ruptured side and ruptured side (post-hCG).

A) 4,945 genes were enriched in the putative unruptured side and 1,942 genes were enriched in the unruptured side post-hCG. B) Samples clustered together by media condition. GO analysis

for biological processes was completed on genes enriched in the C) putative unruptured side and D) unruptured side using Gene Ontology powered by PANTHER.

Supplementary Tables

Supplementary Table 1. Genes regulating pathway analysis on unique and overlapping terms from differential expression analysis within each follicle half.

Supplementary Data

Supplementary Data 1. Source data behind the graphs in the manuscript.

Supplementary Data 2. Complete list of differentially expressed genes between putative ruptured and putative unruptured sides.

Supplementary Data 3. Complete list of differentially expressed genes between ruptured and unruptured sides post-hCG.

Supplementary Data 4. Complete list of differentially expressed genes between putative unruptured and unruptured sides.

Supplementary Data 5. Complete list of differentially expressed genes between putative ruptured and ruptured sides.

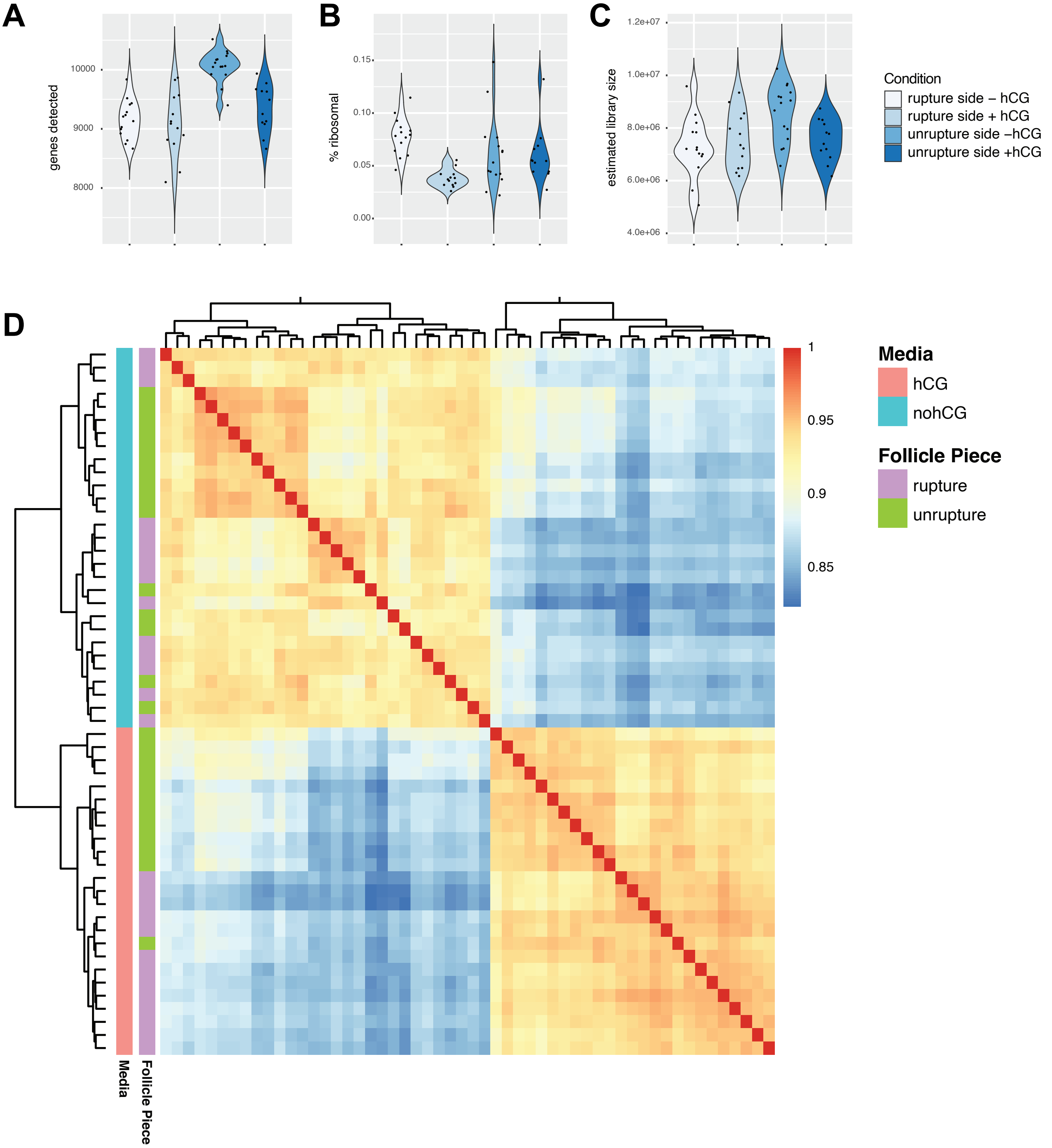

Supplementary Figure 1. All sequenced samples were of high quality.
All sequenced samples had similar numbers of A) genes detected, B) % ribosomal content, and C) estimated library size. D) Samples cluster together based on media conditions (hCG and no hCG).

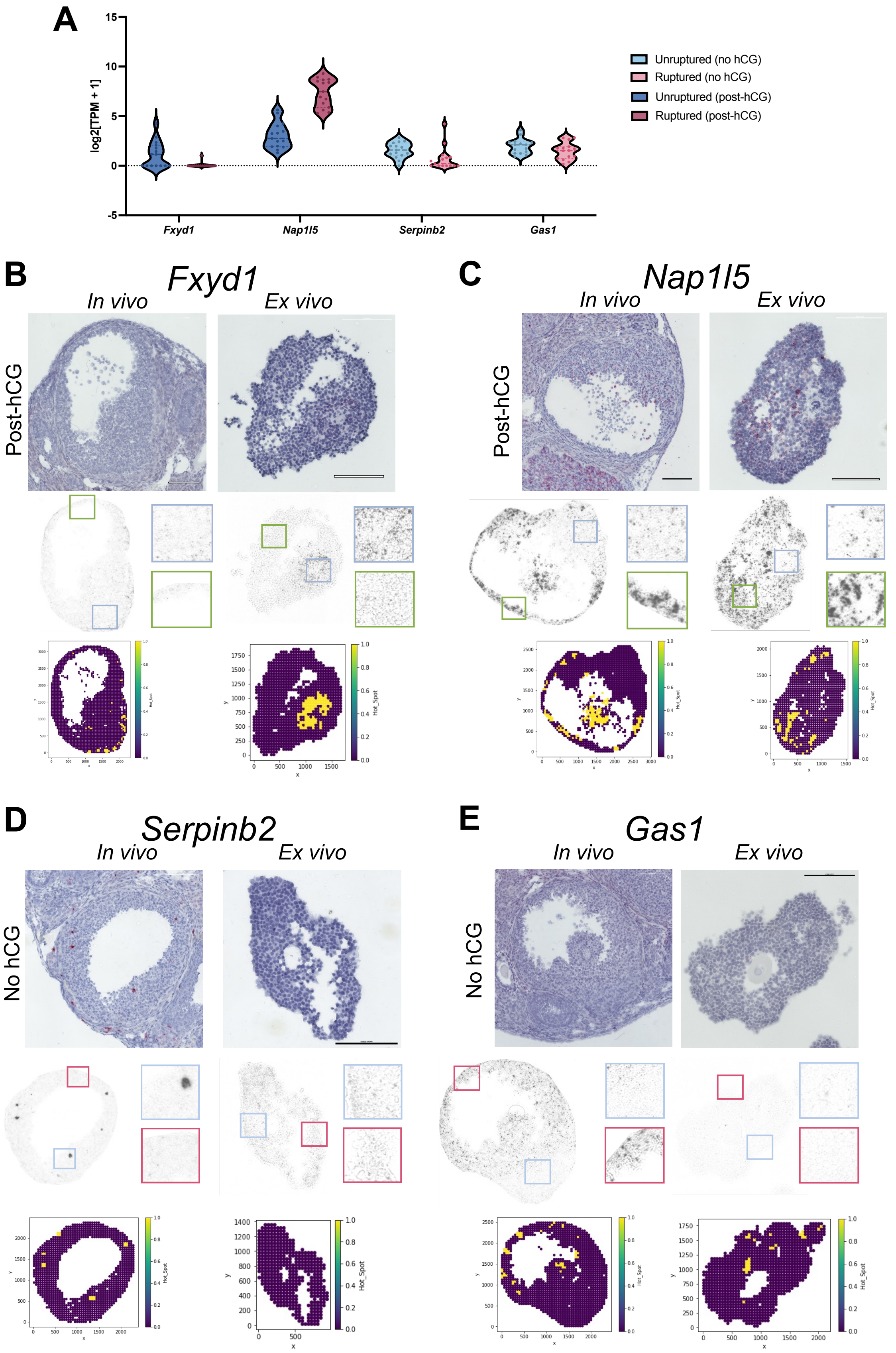

Supplementary Figure 2. RNAscope *in situ* hybridization assay demonstrates consistent expression patterns with sequencing results in non-enriched sample groups.

A) Expression levels of top enriched genes within the other media condition. B-D) All four genes exhibited lower expression levels in the media condition that was not shown in Figure 3. The patterns for genes with detectable expression (*Fxyd1* and *Nap1l5*) were consistent with the transcriptomic results.

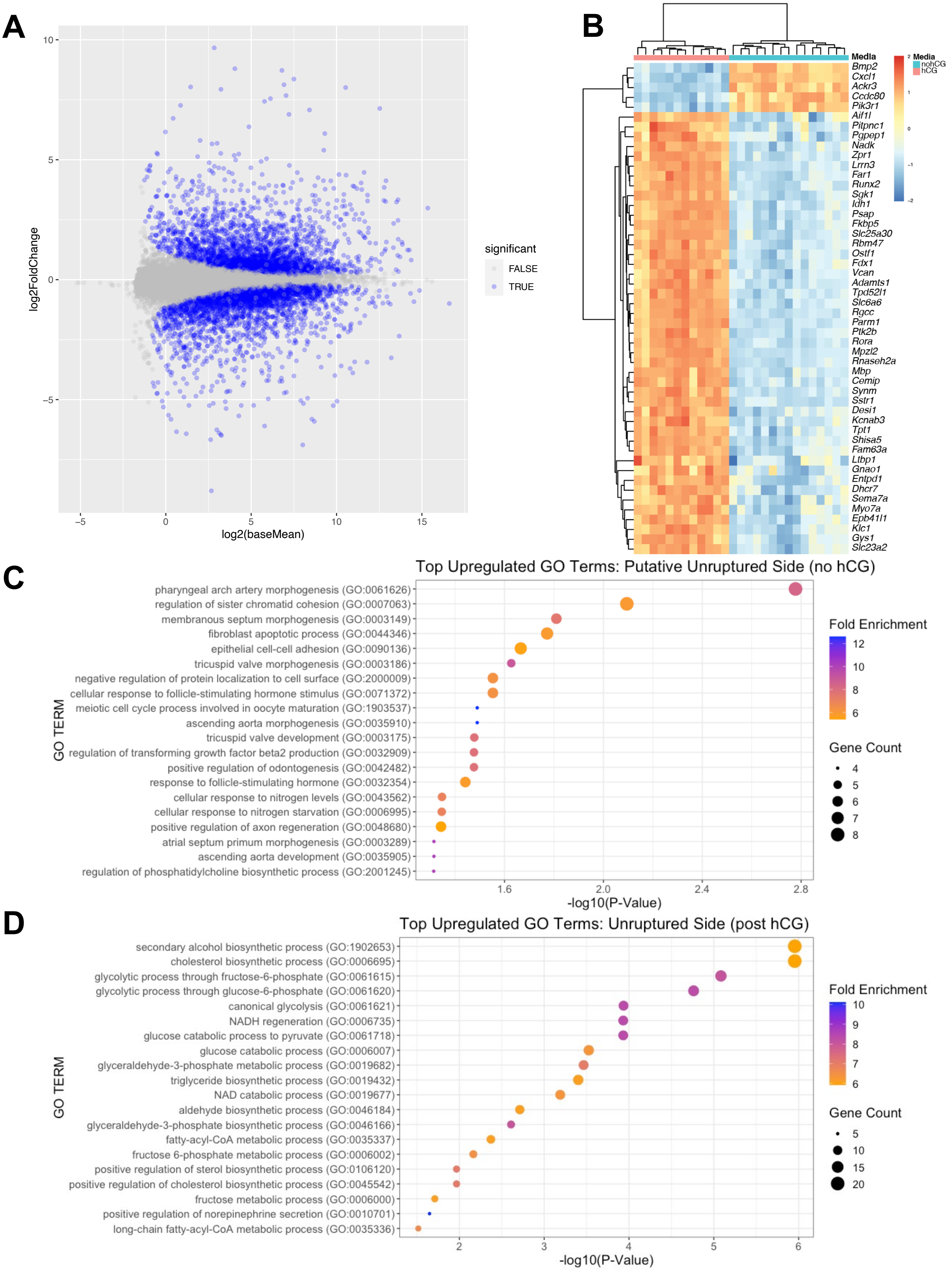

Supplementary Figure 3. Additional comparisons between putative unruptured side and unruptured side (post-hCG).

A) 3,330 genes were enriched in the putative unruptured side and 2,994 genes were enriched in the unruptured side post-hCG. B) Samples clustered together by media condition. GO analysis for biological processes was completed on genes enriched in the C) putative unruptured side and D) unruptured side using Gene Ontology powered by PANTHER.

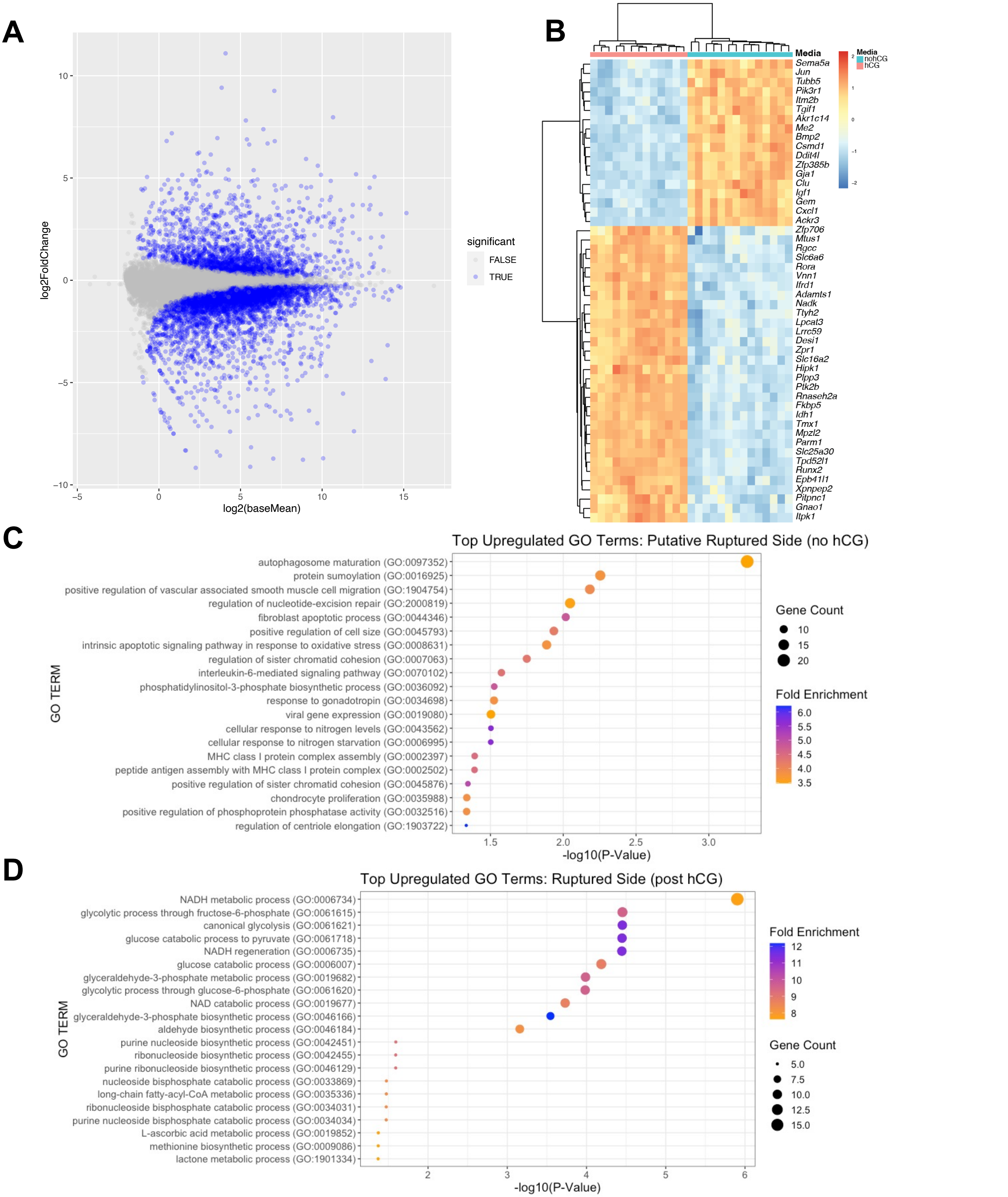

Supplementary Figure 4. Additional comparisons between putative ruptured side and ruptured side (post-hCG).

A) 4,945 genes were enriched in the putative unruptured side and 1,942 genes were enriched in the unruptured side post-hCG. B) Samples clustered together by media condition. GO analysis for biological processes was completed on genes enriched in the C) putative unruptured side and D) unruptured side using Gene Ontology powered by PANTHER.

Supplementary Table 1: Genes regulating pathway analysis on unique and overlapping terms from differential expression analysis within each follicle half.

Putative Unruptured (no hCG) overlap with Unruptured (post-hCG)		
**Putative Unruptured only**	**Overlapping**	**Unruptured only**
662 genes	396 genes	689 genes
**Supramolecular Fiber Organization:** *Col14a1, Flii, Col12a1, Dpt, Slain2, Thsd4, Loxl2, Cnn2, Cdc42, Serpinh1, Ripk1, Rac1, Zbed3, Tmod1, Crtap, Gsn, Tpm3, Tpm2, Shroom3, Serpinb8, Rhoa, Cd2ap, Fkbp1a, Mfap4, Eppk1, Evl, Myl9*<br><br>**Cell Proliferation:** *Cdkn1c, Bmp4, Sfrp1, Dusp10, Erbb2, Eppk1, Scn5a, Prkd1, Mcc, Hras, Nme1, Lims1*	**Collagen Organization:** *Col16a1, Lum, Mmp2, Capg, Nid1, Nid2, Col1a1, Col3a1, Ccdc80, Bmp1, Col1a2, Flrt2, Col5a, Col4a1, Col5a2, Col8a1, Tgfbi, Ddr2*<br><br>**Integrin Signaling:** *Cd63, Lama2, Flna, Emp2, Lamb1, Timp1, Nid1*	**Cellular Respiration:** *Ndufa13, Ndufb8, Ndufb7, Ndufa11, Cox4i1, Cox4i2, Ndufb3, Ndufb2, Cox7a2, Uqcr11, Cox6a1, Cox5b, Cox7c, Uqcrh, Ndufv3, Ndufv1, Cox8a, Ndufa7, Ndufa6, Ndufa5, Mdh2, Ndufa2, Ndufa1, Cox6c, Cox6b1*<br><br>**Glycolytic processes:** *Ldha, Tpi1, Pkm, Pgk1, Aldoc, Eno1, Eno2, Gapdh, Pfkm, Hk1*
**Putative Ruptured (no hCG) overlap with Ruptured (post-hCG)**		
**Putative Ruptured only**	**Overlapping**	**Ruptured only**
851 genes	190 genes	398 genes
**Cholesterol Metabolism:** *Sqle, Nsdhl, Mvk, Insig1, Pmvk, Msmo1, Mvd, Hmgcr, Hsd17b7, Tm7sf2, Fdft1*<br><br>**Mitotic Processes:** *Stil, Plk2, Plk1, Kif23, Aurkb, Mzt1, Ndc80, Cenpe, Tpx2, Incenp, Kifc, Prc1, Dlgap5, Clasp2*<br><br>**DNA Damage Response:** *Pidd1, Cradd, Plk2, Myo6, Sesn2, Atrx, Mdm2, Rps27l, Gtse1*	**Axon/Neural projection:** *Cyfip2, Notch2, Epha4, Nell1, Sema7a, Alcam, Sema3c, Dag1, Plxna1, Sema4f*<br><br>**Amino Acid Transport:** *Slc6a6, Slc7a6, Slc7a8, Slc3a2, Slc38a5*	**Protein Catabolism:** *Ubb, Psme3, Ptk2b, Ube3a, Ctsc*<br><br>**Oxidative Processes:** *Acadvl, Aplp2, Plin5, Chchd4, Lox, Loxl3*
