# Supplementary material for: Follicle-intrinsic and spatially distinct molecular programs drive follicle rupture and luteinization during ex vivo mammalian ovulation
Source: Commun Biol. 2024 Oct 23;7:1374. doi: 10.1038/s42003-024-07074-9 (PMC11500180; doi:10.1038/s42003-024-07074-9)
Supplement: Supplementary file 3 — Description of Additional Supplementary Files [file 42003_2024_7074_MOESM3_ESM.pdf]

Description of Additional Supplementary Files

File name: Supplementary Data 1
Description: Source data behind the graphs in the manuscript.

File name: Supplementary Data 2
Description: Complete list of differentially expressed genes between putative ruptured and putative unruptured sides.

File name: Supplementary Data 3
Description: Complete list of differentially expressed genes between ruptured and unruptured sides post-hCG.

File name: Supplementary Data 4
Description: Complete list of differentially expressed genes between putative unruptured and unruptured sides.

File name: Supplementary Data 5
Description: Complete list of differentially expressed genes between putative ruptured and ruptured sides.
