# Supplementary material for: Follicle-intrinsic and spatially distinct molecular programs drive follicle rupture and luteinization during ex vivo mammalian ovulation
Source: Commun Biol. 2024 Oct 23;7:1374. doi: 10.1038/s42003-024-07074-9 (PMC11500180; doi:10.1038/s42003-024-07074-9)
Supplement: Supplementary file 1 — Review file [file 42003_2024_7074_MOESM1_ESM.pdf]

nature portfolio

Peer Review File

Follicle-intrinsic and spatially distinct molecular programs drive follicle rupture and luteinization during ex vivo mammalian ovulation

Corresponding Author: Dr Francesca Duncan

Version 0:

Reviewer comments:

Reviewer #1

(Remarks to the Author)
Overall, this work represents an extremely thorough transcriptional analysis of an in vitro model of a large follicle that is poised to "ovulate" in vitro in response to administration of an LH surge analog of hCG. Moreover, the authors test the before and after hCG question too. Uniquely, the authors have divided the follicle into a base and apical or unruptured and ruptured side prior to the completing the analyses. The major finding of the study is that gene expression differs for these two regions. The expression is also linked to prior in vivo analyses and regional differences that are well documented as evidenced by the large number references provided. These references document many of the findings seen here are already established in vivo.

Several concerns exist in the interpretation and the presentation of the results.
1. The title needs to include the fact this is an in vitro or ex vivo model. Acknowledging this will not detract from the work, but provide the reader with better understanding of the work from the outset.
2. The authors point to the fact that many of responses are identical to the in vivo situation, and in doing so make the point that these are done in the absence of the natural ovarian stroma, immune cells , changes in blood flow. While true some caution in over interpretation of the data is warranted as it is possible that some of the changes observed may still be an artifact of the culture conditions.
3. The division of the follicle into unruptured and ruptured sides is a simplified view and one could envision that had the authors further divided the follicle perimeter, a transitional zone might have emerged and more striking differences at the poles would have been observed.
4. Specific comments
Line 51 "unprecedented" is an overstatement.
Line 133 - "assymetry forms on apical side adjacent to the ovarian surface epithelium in vivo" This leads to the question what causes the assymetry observed in the ex vivo model in the first place?
Line 296 - the assymetric process in vivo are recapituated in vitro even without hCG". The authors suggests this means the processes are fully follicle-intrinsic and independent of ovulatory hormones and ovarian microenvironment. While the former could be true, the later seems a stretch as something in the alginate hydrobead (assymetric positioning of the follicle for instance) might have provided the follicle with a pathway to allow for expansion of the antrum on one side versus the other.
Line 490 - "Ovulatory mechanisms are autonomous to the follicle itself" - the fact that many gene changes occurred that are similar and that the oocyte can be released in vitro does mean that other ovarian cells are not necessary in vivo. The simple fact that the ovarian stroma,the epithelium and vacular systems are not present does not mean that they are not important and that the follicle on its own could break down these components to allow ovulation in vivo.

Reviewer #2

(Remarks to the Author)
Title: Follicle-intrinsic and spatially distinct molecular programs drive follicle rupture and luteinization during mammalian ovulation
Authors: E.J. Zaniker …… B.A. Goods, F.E Duncan
Review: This is an impressive manuscript that describes studies analyzing structural changes and molecular events that occur in association with hormone-induced ovulation and luteinization in isolated follicles in culture. Specifically, the authors focus on structural and molecular differences that occur in the distinct ruptured versus the non-ruptured surfaces of ovulated follicles. The manuscript contains a wealth of informative imaging and molecular analyses that provide clear evidence that isolated follicles can be cultured and stimulated to undergo antral development and rupture in response to hormones. The

data provide clear evidence that antral follicles do not depend on an intact ovary for hormones to induce the specific ovulation and luteinization processes. The combination of state-of-the-art follicle cultures, elegant imaging and molecular analyses provides and important contribution to our understanding of the molecular events underlying the process of ovulation and luteinization.
The abundance of molecular analyses identify specific genes associated with the ruptured versus non-ruptured surfaces of the ovulating follicle. The studies have been carefully done and provide novel evidence for the unique functions of the surface epithelium that ruptures to release the oocyte compared to the remainder of the follicle that forms the steroidogenic corpus luteum. These studies unequivocally document that an ovulating follicle is a functional entity in and of itself. This is an exciting contribution of our understanding of what regulates distinct events in the ovulating follicle.
Specific comments:
The text needs careful editing. The use of some words seems inappropriate, and/or overly exaggerates the significance of the topic. For example:
Line 38 ….”the unruptured remnant…… Perhaps refer to the unruptured remaining surface of the ovary
Line 48 …”despite separation from intra-ovarian cues”, such as …..
Line 50 …. on the unruptured side of the follicle
Line 51 …unprecedented insights into novel follicle–inherent….. This is a bit over the top.
Line 52 … perhaps rupture of follicular surface is more accurate than “ovulation”
Line 54 .. This first sentence of the Introduction of the manuscript needs to be completely rewritten. It is inaccurate and combines two different concepts: 1) the actual process of ovulation and 2) the fact that ovulation is essential for fertility. The process of ovulation involves the extrusion of the COC to the surface of the ovary but does not lead immediately to the release of the COC from the ovarian surface into the Fallopian tube. Rather the Fallopian tube sweeps over the ovarian surface and collects the COC attached to the surface of the ovary by the activity of ciliated cells.
Line 60: What does “supported by luteinization” refer to?
Line 63: “discontinuation” does not fit here. Improving “our” understanding
Line 85: what does “temporary” refer to?
Lines 87-88: This sentence does not make sense.
Lines103-104: these lines are misleading. The ovulatory rupture site is very small and the COC remains adherent to the ovarian surface where it is ultimately picked up by the fibria of the oviduct.
Line 115: Specifically (not ultimately), we found …
Lines 141-142 do not make sense.
These are just a few examples.

Methods/Results:
How many follicles were used in each set of experiments?
Line 568 is the only place that spefically indicates 92 follicles were analyzed for imaging analyses of asymmetry. How about for the RNAseq analyses
and other imaging analyses in Figures 1-4?

Figures: The Figures are clearly presented and artfully done, however some of the descriptions of the data in the figures could be presented more clearly.

Minor comments
Figure 1, Panel A: the black asterisk in the antral follicle is difficult to see.
The Ex Vivo images need to be labeled more clearly.
Panel G: Is the central dark area an unovulated COC? Perhaps this could be clarified. Or perhaps there is a better image.
What do the pink and blue arrows identify in panels C and F. Panel D: Lines 1233-1234 in the text do not relate to panel D clearly.
Line 1240: Were any COC genes analyzed to confirm COC expansion in this model?
Figure 2: Line 1248 (remove “in the”); luteinization 48 hours post-ovulation
Lines 1256-1258: Figure 2B: brightfield images not clear and not clearly labeled
Figure 3:Lines 1277-1278 are not clear. What happened to the COCs?
Figure 8: Nice summary but more information is needed in the Figure Legend.

Discussion

Line 379: Perhaps stated better as “ The asymmetry of follicles undergoing ovulation” … The text needs to be carefully edited.

Reviewer #3

(Remarks to the Author)
In this manuscript, Zanikar et al. performed light microscopy and RNA-seq analysis on an ex vivo ovulation model to study the ruptured and unruptured sides of the ovarian follicle wall during follicle maturation and ovulation. Using a machine-learning method, the authors identified and microdissected the putative ruptured and unruptured sides of the model ovarian follicular wall in the preovulatory stage (before the rupture actually happens). The authors then performed RNA-seq on the microdissected tissues to find molecular differences between the ruptured and unruptured sides of the follicle wall both

before and after ovulation. From these analyses, they not only confirmed several previous findings by identifying known spatially-patterned genes and pathways enriched in the (putative) ruptured or unruptured sides of the follicle wall but also found several new and interesting genes and broad functional pathways associated with luteinization and rupture itself.

Previous studies using single-cell sequencing of the ovary have shown greater than expected cellular diversity but could not identify genes or pathways spatially restricted to the ruptured or unruptured sides of the wall. This study fills that gap by illuminating these spatial differences; however, it lacks single-cell resolution. Recent advances in high-resolution spatial transcriptomic technologies provide another means to study this spatial asymmetry with complete ovarian complexity in vivo.

Overall, the study is well designed, beautifully implemented, and properly analyzed. The work provides new insights into the molecular differences and pathways of follicle maturation and ovulation in mice and is likely to influence thinking in the field. The gene expression data and the differential gene expression results generated in this work are likely to benefit other researchers in the field.

Addressing the following concerns would improve the manuscript and strengthen the results:

1. Lines 195-201: Do all ex vivo follicles cultured in hCG go through follicle rupture? The gene expression-based follicle clustering presented in the manuscript shows consistent molecular patterns within ruptured and unruptured sites in almost all follicles for both no-hCG and post-hCG treated follicles. The live/dead cell assays also show significant differences. However, in an in vivo system, only one follicle from a wave of growing follicles has ovulatory capacity and is selected for ovulation, while others undergo luteinization. Can the authors discuss this difference between the in vivo and ex vivo systems and the reason behind the differences in follicle selection (if any) in the ex vivo system?

2. Lines 243-260: The RNA-FISH validation performed to check asymmetric expression of genes identified from ex vivo analysis is not very clear in the in vivo system. The authors do acknowledge that the asymmetry in some cases is more dramatic in the ex vivo follicles. These asymmetric gene expression patterns are the highlight of the paper, and therefore more imaging-based validation with quantitative comparisons of RNA-FISH or immunostaining signals between ruptured and unruptured walls would significantly strengthen the confidence in these results. Concerns:
a) In Fig. 4B, the presence of Fxyd transcripts on the unruptured side of the wall is very clear in ex vivo but not very clear in vivo.
b) In Fig. 4D, Serpinb2 expression seems very sparse in some granulosa cells (not certain) of the unruptured side in ex vivo follicles but seems to be present in ovarian stromal cells or theca cells in vivo.
c) In Fig. 4E, hotspots of Gas1 are visible in the mural granulosa layer all around the follicle except in the part with a very thin and potentially putative ruptured wall (apex of the follicle on the left), which is the opposite of what is seen in the ex vivo system.

3. Line 245-246 (related to the previous comment): For in vivo validation of asymmetric patterns, the text states that the putative unruptured side (light pink arrow and box) is selected opposite to the thinning region of the ruptured side (light blue arrow and box). However, that is not the case in images with Fxyd1 and Gas1 staining in Fig 4B & 4E.

4. Lines 265-300: Gene ontology analysis of genes enriched in ruptured or unruptured walls revealed several novel asymmetric pathways. Visualization of these pathway activities in the in vivo system would really strengthen the paper. Using the genes associated with these pathways, the authors should estimate and visualize pathway module scores on spatial transcriptomic datasets from ovulating ovaries published recently (PMID: 38826447 and PMID: 37662215). The authors could additionally select apex and the base (putative ruptured vs unruptured sites) of growing and preovulatory follicles and do a quantitative comparison of the module scores. This additional analysis could significantly strengthen the novel asymmetric pathway results and would be of value to the field.

5. Lines 265-300 (Related to the previous comment): Were the theca cells retained in the follicles during isolation for ex vivo experiments? It is not very clear if granulosa cells or theca cells are driving some of these asymmetric molecular patterns in the follicle wall. Asymmetry in several pathways, such as angiogenesis, muscle contraction, apoptosis, and even in signaling pathways such as NOTCH, WNT, and BMP, could be attributed to granulosa cells alone, theca cells alone, or even both. The authors can address this limitation of the technique by analyzing high-resolution spatial transcriptomics data sets discussed in the previous comment. Identification of the cell types for these functional pathways would not only strengthen this work but would influence thinking in the field on how to use these discoveries for reproductive medicine.

6. Lines 528-529: Were secondary follicles collected from multiple animals for every ex vivo experiment described in the manuscript? Details regarding how many animals were used should be included in future captions and/or the methods section.

Author Rebuttal letter:

Dear Dr. Mengtan Xing,

We thank the Editors for the opportunity to submit a revised version of our manuscript to Communications Biology, now entitled “Follicle-intrinsic and spatially distinct molecular programs drive follicle rupture and luteinization during ex vivo mammalian ovulation.” Our findings demonstrate that follicle-inherent asymmetrical pathways in ex vivo ovulatory follicles

can be harnessed to identify novel pathways that drive follicle rupture and luteinization. Our manuscript is tightly aligned with the scope of the special issue on Biology of Reproduction.

We are grateful to the reviewers for their insightful comments and suggestions, which we have fully integrated and which have strengthened the manuscript. We have modified the interpretation and presentation of results, completed additional replicates of RNAscope in situ hybridization, and incorporated analyses from recent single cell and spatial transcriptomics datasets. We have provided a point-by-point response to the reviewer comments below and have included both a clean version and one with highlighted changes for ease of review. Line numbers corresponding to the clean version are noted when relevant.

Reviewers' comments:

Reviewer #1 (Remarks to the Author):
Overall, this work represents an extremely thorough transcriptional analysis of an in vitro model of a large follicle that is poised to "ovulate" in vitro in response to administration of an LH surge analog of hCG. Moreover, the authors test the before and after hCG question too. Uniquely, the authors have divided the follicle into a base and apical or unruptured and ruptured side prior to the completing the analyses. The major finding of the study is that gene expression differs for these two regions. The expression is also linked to prior in vivo analyses and regional differences that are well documented as evidenced by the large number references provided. These references document many of the findings seen here are already established in vivo.
Thank you for this summary of our work and for the suggestions, which we have responded to below.

Several concerns exist in the interpretation and the presentation of the results.
Comment 1: The title needs to include the fact this is an in vitro or ex vivo model. Acknowledging this will not detract from the work, but provide the reader with better understanding of the work from the outset.
Thank you for this suggestion. We have changed the manuscript title to: “Follicle-intrinsic and spatially distinct molecular programs drive follicle rupture and luteinization during ex vivo mammalian ovulation.”

Comment 2: The authors point to the fact that many of responses are identical to the in vivo situation, and in doing so make the point that these are done in the absence of the natural ovarian stroma, immune cells, changes in blood flow. While true some caution in over interpretation of the data is warranted as it is possible that some of the changes observed may still be an artifact of the culture conditions.
We selected our ex vivo model because it recapitulates key structural, functional, and molecular signatures of in vivo ovulation1. Our current study demonstrates that many of the core features of molecular asymmetry during ovulation are conserved in our ex vivo model. We do not believe this is an artifact of our culture conditions because the ability of follicles to undergo follicular rupture in response to ovulatory cues has been observed in several different systems2. Nevertheless, we agree that the ovarian stroma, immune cells, and vasculature likely play important roles in in vivo ovulation.
Citations:
1. Zhang, J., Goods, B. A., Pattarawat, P., Wang, Y., Haining, T., Zhang, Q., Shalek, A. K., Duncan, F. E., Woodruff, T. K., Xiao, S. An ex vivo ovulation system enables the discovery of novel ovulatory pathways and nonhormonal contraceptive candidates, Biology of Reproduction, 108(4), 629–644, (2023). https://doi.org/10.1093/biolre/ioad009
2. Zaniker, E. J., Hashim, P. H., Gauthier, S., Ankrum, J. A., Campo, H., and Duncan, F. E. Three-Dimensionally Printed Agarose Micromold Supports Scaffold-Free Mouse Ex Vivo Follicle Growth, Ovulation, and Luteinization. Bioengineering 11(7), 719, (2024).

We have revised the manuscript text to acknowledge the importance of the ovarian microenvironment and alleviate potential concerns regarding culture artifacts.

Line 543-553: Our findings demonstrate that there are follicle-intrinsic mechanisms that promote asymmetric antrum formation, and this does not appear to be an artifact of the culture conditions as it is observed in non-encapsulated ex vivo systems as well178. Nevertheless, extrinsic factors from the ovarian microenvironment also play a clear role in driving this asymmetry in vivo. The ovarian microenvironment may play a particularly important role in establishing the directionality of the asymmetry and to ensure release of the egg at the correct time and place to provide for optimal fertilization potential through contributions of the stroma, vasculature, and epithelial components12. Novel ex vivo follicle culture strategies that incorporate features of the ovarian microenvironment on a structural or molecular level will be essential tools to further elucidate the factors required for optimal follicular rupture and ovulation178,179.

Comment 3: The division of the follicle into unruptured and ruptured sides is a simplified view

and one could envision that had the authors further divided the follicle perimeter, a transitional zone might have emerged and more striking differences at the poles would have been observed.
We divided the follicles into two distinct regions based on the resolution of our predictive model. Our concern about sampling the most distant regions at opposite poles is that we would potentially miss important tissue regions that contribute to the ovulatory process. Our approach in fact yielded several thousand differentially expressed genes demonstrating the robustness of follicle asymmetry during ex vivo ovulation. We have addressed the point raised in the manuscript text:

Line 553-561: In addition, though our study used a machine learning guided microdissection technique to identify follicle sides, there are limitations to our approach. Ovulatory follicles are complex structures that may have a gradient of molecular and morphologic changes that cannot be captured by microdissection of the follicle wall grossly into two discrete sections. Future studies incorporating spatial transcriptomics or whole-ovary immunofluorescence with tissue clearing may provide a higher degree of spatial resolution than was possible with our technique135,180-182. Despite this limitation, we still identified thousands of differentially expressed genes and pathways of biological interest demonstrating that follicle asymmetry during ex vivo ovulation is a robust phenotype.

Specific comments
Comment 4a: Line 51 "unprecedented" is an overstatement.
This line (now line 50-52) now reads: This study yielded unprecedented insights novel insights into novel follicle-inherent, spatially-defined pathways that underlie ovulation follicle rupture, which may further understanding of ovulation physiology and advance women's health.
Comment 4b: Line 133 - "assymetry forms on apical side adjacent to the ovarian surface epithelium in vivo" This leads to the question what causes the assymetry observed in the ex vivo model in the first place?
Line 296 - the assymetric process in vivo are recapituated in vitro even without hCG". The authors suggests this means the processes are fully follicle-intrinsic and independent of ovulatory hormones and ovarian microenvironment. While the former could be true, the later seems a stretch as something in the alginate hydrobead (assymetric positioning of the follicle for instance) might have provided the follicle with a pathway to allow for expansion of the antrum on one side versus the other.
The two above points are related to the same question of how asymmetry is formed ex vivo. It is not currently known what forces specifically drive this asymmetry (either in vivo or ex vivo). Based on our observations, it does not appear that position within the alginate bead specifies direction of antrum formation. When we encapsulate follicles, we try to position them at the center of the alginate bead and the forces on the follicle should be equally distributed. There does not appear to be any clear pattern of antrum formation towards the center of the bead or away from the bead. What we do know is that formation of asymmetry appears to be essential for formation of a follicle rupture site, both in vivo and ex vivo. It is likely that the ovarian microenvironment drives proper positioning of the asymmetry such that the rupture site forms adjacent to the ovarian surface to allow for extrusion of the COC. In absence of this microenvironment, the directionality of the asymmetry appears to be random. It is also possible that there is a degree of cellular memory within the ovarian context, which has been previously demonstrated in aging studies and could drive the direction of asymmetry1. The factors that drive asymmetry of antrum formation and subsequent ovulation ex vivo is an active area of study in our lab and we are in the process of developing novel ex vivo methods that better address this question by integrating an in vivo-like biomechanical support structure that drives follicle asymmetry in a particular direction2.

Citations:
1. Hirshfeld-Cytron J. E., Duncan F. E., Xu M., Jozefik J. K., Shea L. D., Woodruff T. K. Animal age, weight and estrus cycle stage impact the quality of in vitro grown follicles. Human Reproduction. 26(9), 2473-85 (2011). doi: 10.1093/humrep/der183
2. Zaniker, E. J., Hashim, P. H., Gauthier, S., Ankrum, J. A., Campo, H., and Duncan, F. E. Three-Dimensionally Printed Agarose Micromold Supports Scaffold-Free Mouse Ex Vivo Follicle Growth, Ovulation, and Luteinization. Bioengineering 11(7), 719, (2024).

We have included the following text to the manuscript to address both of the above questions:

Line 538-546: Although it is not yet known what drives the asymmetry within antral follicles, this asymmetry is essential for formation of a follicle rupture site. When the follicle is situated within the ovarian microenvironment, there are likely biomechanical and molecular signals that drive the asymmetry in a particular direction to facilitate follicle rupture adjacent to the ovarian surface as a prerequisite for COC release. When the follicle is removed from this environment, we have demonstrated that asymmetry is maintained and similarly dictates rupture of the follicle. Our

findings demonstrate that there are follicle-intrinsic mechanisms that promote asymmetric antrum formation, and this does not appear to be an artifact of the culture conditions as it is observed in non-encapsulated ex vivo systems as well178.

Comment 4c: Line 490 - "Ovulatory mechanisms are autonomous to the follicle itself" - the fact that many gene changes occurred that are similar and that the oocyte can be released in vitro does mean that other ovarian cells are not necessary in vivo. The simple fact that the ovarian stroma,the epithelium and vacular systems are not present does not mean that they are not important and that the follicle on its own could break down these components to allow ovulation in vivo.
Thank you for this comment. As noted in response to Comment 2 and 4b, we do not intend to imply that the stroma, epithelium, and vasculature are not important for ovulation. Instead, we are proposing that key drivers of asymmetry during follicle rupture can occur in their absence with preserved molecular signatures1.

Citations:
1. Zhang, J., Goods, B. A., Pattarawat, P., Wang, Y., Haining, T., Zhang, Q., Shalek, A. K., Duncan, F. E., Woodruff, T. K., Xiao, S. An ex vivo ovulation system enables the discovery of novel ovulatory pathways and nonhormonal contraceptive candidates, Biology of Reproduction, 108(4), 629–644, (2023). https://doi.org/10.1093/biolre/ioad009

We have added and modified the following text to the manuscript to clarify this point:

Line 546-551: Nevertheless, extrinsic factors from the ovarian microenvironment also play a clear role in driving this asymmetry in vivo. The ovarian microenvironment may play a particularly important role in establishing the directionality of the asymmetry and to ensure release of the egg at the correct time and place to provide for optimal fertilization potential through contributions of the stroma, vasculature, and epithelial components12.

Line 490 (Now line 562-564): In summary, our study demonstrated that there are a series of core ovulatory mechanisms which are autonomous to the follicle itself and can occur even in the absence of the ovarian context. do not require ovarian cells to complete the process of tissue breakdown.

Reviewer #2 (Remarks to the Author):
Title: Follicle-intrinsic and spatially distinct molecular programs drive follicle rupture and luteinization during mammalian ovulation
Authors: E.J. Zaniker …… B.A. Goods, F.E Duncan

Review: This is an impressive manuscript that describes studies analyzing structural changes and molecular events that occur in association with hormone-induced ovulation and luteinization in isolated follicles in culture. Specifically, the authors focus on structural and molecular differences that occur in the distinct ruptured versus the non-ruptured surfaces of ovulated follicles. The manuscript contains a wealth of informative imaging and molecular analyses that provide clear evidence that isolated follicles can be cultured and stimulated to undergo antral development and rupture in response to hormones. The data provide clear evidence that antral follicles do not depend on an intact ovary for hormones to induce the specific ovulation and luteinization processes. The combination of state-of-the-art follicle cultures, elegant imaging and molecular analyses provides and important contribution to our understanding of the molecular events underlying the process of ovulation and luteinization.

The abundance of molecular analyses identify specific genes associated with the ruptured versus non-ruptured surfaces of the ovulating follicle. The studies have been carefully done and provide novel evidence for the unique functions of the surface epithelium that ruptures to release the oocyte compared to the remainder of the follicle that forms the steroidogenic corpus luteum. These studies unequivocally document that an ovulating follicle is a functional entity in and of itself. This is an exciting contribution of our understanding of what regulates distinct events in the ovulating follicle.
We appreciate this enthusiastic evaluation of our work and the suggestions provided for improvement which we have addressed below.

Specific comments:
The text needs careful editing. The use of some words seems inappropriate, and/or overly exaggerates the significance of the topic. For example:
Comment 1a: Line 38 ….”the unruptured remnant…… Perhaps refer to the unruptured remaining surface of the ovary
This line (now line 38-39) now reads: In parallel, the residual follicle wall unruptured remnant undergoes differentiation differentiates into a progesterone-producing corpus luteum.

Comment 1b: Line 48 …"despite separation from intra-ovarian cues", such as …..
This line (now line 46-48) now reads: Our model validated molecular patterns consistent with known ovulation biology even though this process occurred in the absence of intra-ovarian cues. the ovarian stroma, vasculature, and immune cells.

Comment 1c: Line 50 …. on the unruptured side of the follicle
This line (now line 48-50) now reads: We further identified previously unappreciated pathways including amino acid transport and Jag-Notch signaling on the ruptured side and glycolysis, metal ion processing, and IL-11 signaling on the unruptured side of the follicle.

Comment 1d: Line 51 …unprecedented insights into novel follicle–inherent….. This is a bit over the top.
Line 52 … perhaps rupture of follicular surface is more accurate than "ovulation"
These lines (now line 50-52) now read: This study yielded unprecedented insights novel insights into novel follicle-inherent, spatially-defined pathways that underlie ovulation follicle rupture, which may further understanding of ovulation physiology and advance women's health.

Comment 1e: Line 54 .. This first sentence of the Introduction of the manuscript needs to be completely
rewritten. It is inaccurate and combines two different concepts: 1) the actual process of ovulation and 2) the fact that ovulation is essential for fertility. The process of ovulation involves the extrusion of the COC to the surface of the ovary but does not lead immediately to the release of the COC from the ovarian surface into the Fallopian tube. Rather the Fallopian tube sweeps over the ovarian surface and collects the COC attached to the surface of the ovary by the activity of ciliated cells.
This line (now line 54-55) now reads: Ovulation involves the coordinated release of the cumulus oocyte complex (COC) from the ovarian follicle, into the fallopian tube and which is essential for human fertility.

Comment 1f: Line 60: What does "supported by luteinization" refer to?
This line (now line 57-60) now reads: In addition, ovulation represents an important biological process that can be targeted for the development of non-hormonal contraceptives by identifying ways to block follicle rupture during ovulation without interfering with hormone production during the luteal phase. the hormonal axis supported by luteinization.

Comment 1g: Line 63: "discontinuation" does not fit here. Improving "our" understanding
This line (now line 60-64) now reads: Such contraceptives would represent a significant advance given that currently available hormonal contraceptives exhibit side effects ranging from mood changes to increased risk of blood clots, resulting in non-use or mis-use, or discontinuation5,6. Improving our understanding of ovulation on a molecular level, therefore, would have important implications for human health and fertility.

Comment 1h: Line 85: what does "temporary" refer to?
This line (now line 84-87) now reads: These cells form a temporary transient endocrine structure called the corpus luteum that supports the hormonal axis of the ovulatory cycle and signals change in the uterine lining to stimulate glandular secretion that supports early embryonic development in the case of pregnancy21,48,49.

Comment 1i: Lines 87-88: This sentence does not make sense.
This line (now line 87-89) now reads: Regression of the corpus luteum following ovulation triggers leads to a decline in progesterone levels, which induces shedding of the endometrial uterine lining of the uterus during menstruation the menstrual phase of the uterine cycle50-52.

Comment 1j: Lines103-104: these lines are misleading. The ovulatory rupture site is very small and the COC remains adherent to the ovarian surface where it is ultimately picked up by the fibria of the oviduct.
This line (now line 102-105) now reads: Smooth muscle-like contractions are also spatially restricted to the unruptured side of the follicle wall, which is thought to exert a mechanical force that contributes to follicle rupture and COC release to the ovarian surface where it is ultimately picked up by the fimbria of the oviduct pulls apart the ruptured site and facilitate COC release33-35,39,43-47,72-74.

Comment 1k: Line 115: Specifically (not ultimately), we found …
This line (now line 116-118) now reads: Specifically, Ultimately, we found spatial differences in the luteinization capacity and several novel asymmetric pathways within the ovulatory follicle wall, demonstrating that the two sides of the follicle wall are functionally and molecularly distinct.

Comment 1l: Lines 141-142 do not make sense.
This line (now line 141-144) now reads: This morphological marker Regions of reduced optical density, identified by heat maps in the machine learning model, was used to guide decisions on microdissection to separate the putative ruptured and putative unruptured sides with the COC removed (Figure 1D-F).

Comment 1m: These are just a few examples.
Thank you for these suggestions. In addition, we have thoroughly reviewed the manuscript to ensure clarity.

Methods/Results:
Comment 2: How many follicles were used in each set of experiments?
Line 568 is the only place that spefically indicates 92 follicles were analyzed for imaging analyses of asymmetry. How about for the RNAseq analyses
and other imaging analyses in Figures 1-4?
We have added sample size details to all figure legends and the manuscript text.

Figures:
Comment 3: The Figures are clearly presented and artfully done, however some of the descriptions of the data in the figures could be presented more clearly.
We have reviewed and revised all figure legends for clarity.
Minor comments
Comment 4a:
Figure 1, Panel A: the black asterisk in the antral follicle is difficult to see.
We have moved the placement of the asterisks in Figure 1A to make them easier to see.

The Ex Vivo images need to be labeled more clearly.
We have modified the labels for the ex vivo images.

Panel G: Is the central dark area an unovulated COC? Perhaps this could be clarified. Or perhaps there is a better image.
The central dark area is a COC. At the 12-hour time point that we use, the follicle has ruptured but the COC is not always fully extruded from within the follicle walls. We have replaced the image used in Figure 1G to one that is clearer.

What do the pink and blue arrows identify in panels C and F. Panel D: Lines 1233-1234 in the text do not relate to panel D clearly.
The pink arrows indicate the ruptured side and the blue arrows indicate the unruptured side, which is now reflected in the figure legend. Lines 1233-1234 were removed from the figure legend.

Line 1240: Were any COC genes analyzed to confirm COC expansion in this model?
No COC genes were analyzed as COCs were removed from our microdissected follicles prior to sequencing. As such, we would not be able to use sequencing data to assess COC expansion.

To further clarify these points, the following lines of text were revised:

Lines 1367-1370: Based on morphologic hallmarks of the putative ruptured and unruptured sides and guidance from the machine learning model, follicles in the no-hCG condition were microdissected. The COC was removed and excluded so that only the contribution of the follicle was analyzed.

Comment 4b:
Figure 2: Line 1248 (remove “in the”); luteinization 48 hours post-ovulation
This text was removed.

Lines 1378-1379: Figure 2. The unruptured side of the follicle wall exhibits key signs of luteinization in the 48 hours post-ovulation, while the ruptured side exhibits signs of cell death during the same period.

Lines 1256-1258: Figure 2B: brightfield images not clear and not clearly labeled
The labels on the brightfield images in Figure 2B were revised.

Comment 4c:
Figure 3:Lines 1277-1278 are not clear. What happened to the COCs?
For all experiments, COCs were removed when follicles were microdissected and were not included in any downstream analyses.

The following text was revised to improve clarity:
Lines 1406-1408: After 12 hours, we microdissected the follicles to separate the unruptured side from the ruptured side, and COCs were removed and discarded. Only the individual follicle sides were submitted them for bulk RNA-sequencing.
Lines 1367-1370: Based on morphologic hallmarks of the putative ruptured and unruptured sides and guidance from the machine learning model, follicles in the no-hCG condition were microdissected. The COC was removed and excluded so that only the contribution of the follicle was analyzed.

Comment 4d:
Figure 8: Nice summary but more information is needed in the Figure Legend.
The figure legend was updated to the following:

Lines 1483-1490: Distinct regions of the ex vivo follicle wall exhibit functional and molecular differences that both recapitulate known ovulation biology and represent novel pathways that may participate in asymmetric functions during the periovulatory period. These pathways, identified through gene ontology, represent areas of potential biological significance for the processes of ovulation and luteinization. Further analysis identified cell-type specificity of each pathway. Some genes underlying key pathways were enriched primarily in granulosa cells (green), theca cells (orange), or a combination of both cell types (gray). The cumulus-oocyte-complex, which was excluded from our analysis, is shown in black and white.

Discussion
Comment 5: Line 379: Perhaps stated better as “ The asymmetry of follicles undergoing ovulation” … The text needs to be carefully edited.
This line (now line 409-411) has been updated to read: The asymmetry that underlies ovulation of follicles undergoing ovulation in vivo has been described for decades and underlies release of a fertilization-competent oocyte and maintenance of the hormonal axis that supports establishment of pregnancy.

In addition, we have thoroughly reviewed the manuscript to ensure clarity.

Reviewer #3 (Remarks to the Author):

In this manuscript, Zanikar et al. performed light microscopy and RNA-seq analysis on an ex vivo ovulation model to study the ruptured and unruptured sides of the ovarian follicle wall during follicle maturation and ovulation. Using a machine-learning method, the authors identified and microdissected the putative ruptured and unruptured sides of the model ovarian follicular wall in the preovulatory stage (before the rupture actually happens). The authors then performed RNA-seq on the microdissected tissues to find molecular differences between the ruptured and unruptured sides of the follicle wall both before and after ovulation. From these analyses, they not only confirmed several previous findings by identifying known spatially-patterned genes and pathways enriched in the (putative) ruptured or unruptured sides of the follicle wall but also found several new and interesting genes and broad functional pathways associated with luteinization and rupture itself.

Previous studies using single-cell sequencing of the ovary have shown greater than expected cellular diversity but could not identify genes or pathways spatially restricted to the ruptured or unruptured sides of the wall. This study fills that gap by illuminating these spatial differences; however, it lacks single-cell resolution. Recent advances in high-resolution spatial transcriptomic technologies provide another means to study this spatial asymmetry with complete ovarian complexity in vivo.

Overall, the study is well designed, beautifully implemented, and properly analyzed. The work provides new insights into the molecular differences and pathways of follicle maturation and ovulation in mice and is likely to influence thinking in the field. The gene expression data and the differential gene expression results generated in this work are likely to benefit other researchers in the field.

Thank you for this summary of our work and these suggestions. We have incorporated your feedback into our revised manuscript and responded to each comment on a point-by-point basis below.

Addressing the following concerns would improve the manuscript and strengthen the results:

Comment 1:

Lines 195-201: Do all ex vivo follicles cultured in hCG go through follicle rupture? The gene expression-based follicle clustering presented in the manuscript shows consistent molecular patterns within ruptured and unruptured sites in almost all follicles for both no-hCG and post-hCG treated follicles. The live/dead cell assays also show significant differences. However, in an in vivo system, only one follicle from a wave of growing follicles has ovulatory capacity and is selected for ovulation, while others undergo luteinization. Can the authors discuss this difference between the in vivo and ex vivo systems and the reason behind the differences in follicle selection (if any) in the ex vivo system?

In our ex vivo system, around 80% of follicles survive to the antral stage1. Of those surviving follicles, around 80% successfully undergo follicle rupture1. When we isolate follicles, we typically get a yield of 5-10 multilayer secondary follicles per ovary and we only select the healthy follicles with optimal morphology. This selection likely removes some of the follicles that would undergo atresia within the ovary. In addition, the ex vivo system likely rescues some follicles by removing them from exposure to paracrine factors from other maturing follicles, which is a significant driver of follicle selection and atresia in vivo2. Importantly, the follicles that do survive and grow appear to be fully functional, shown by their ability to grow, produce hormones, and produce meiotically competent gametes. It is also important to note that rodents are polyovulatory, unlike humans and other large mammals, meaning multiple follicles mature to the point of ovulation during each cycle.

Citations:
1. Converse, A., Zaniker, E. J., Amargant, F., and Duncan, F. E. Recapitulating folliculogenesis and oogenesis outside the body: encapsulated in vitro follicle growth. Biology of reproduction 108(1), 5-22, (2023).
2. Findlay, J. K., K. R. Dunning, R. B. Gilchrist, K. J. Hutt, Darryl L. Russell, and K. A. Walters. Follicle selection in mammalian ovaries. In The ovary, 3-21. Academic Press, (2019).

To address this point, we have added the following text to the discussion of our manuscript:

Line 617-622: As follicles are cultured individually in our system and were therefore not exposed to paracrine factors from other nearby follicles, we removed the follicle selection and atresia that occurs in vivo91. We completed follicle selection during the follicle isolation process to only select the most optimal follicles for culture, which provided artificial follicle selection and allowed for a yield of more than 80% of follicles that survived and matured to the antral stage91.
Comment 2:
Lines 243-260: The RNA-FISH validation performed to check asymmetric expression of genes identified from ex vivo analysis is not very clear in the in vivo system. The authors do acknowledge that the asymmetry in some cases is more dramatic in the ex vivo follicles. These asymmetric gene expression patterns are the highlight of the paper, and therefore more imaging-based validation with quantitative comparisons of RNA-FISH or immunostaining signals between ruptured and unruptured walls would significantly strengthen the confidence in these results. Concerns:
Thank you for this suggestion. One challenge of modeling asymmetry within the ovary is that we are largely reliant on histologic sections. These sections turn a 3D structure, the ovulatory follicle, into a 2D cross section. It is difficult to ensure that a follicle in a cross section includes representative areas of both the ruptured and unruptured sides. To account for this, we strategically select histologic sections that appear to include both regions within one ovulatory follicle. See below for specific responses and a revised Figure 4.
a) In Fig. 4B, the presence of Fxyd transcripts on the unruptured side of the wall is very clear in ex vivo but not very clear in vivo.
The in vivo section for 4B has been updated to include a clearer representation of follicle halves. The expression still appears substantially lower in vivo, as we discuss briefly in the results section.
b) In Fig. 4D, Serpinb2 expression seems very sparse in some granulosa cells (not certain) of the unruptured side in ex vivo follicles but seems to be present in ovarian stromal cells or theca cells in vivo.
The ex vivo section for 4D has been updated to include a clearer representation of follicle halves. We have also included a new figure providing information on cell type specificity to further elucidate expression patterns of key enriched genes.
c) In Fig. 4E, hotspots of Gas1 are visible in the mural granulosa layer all around the follicle except in the part with a very thin and potentially putative ruptured wall (apex of the follicle on the left), which is the opposite of what is seen in the ex vivo system.
The in vivo section for 4E has been updated to include a clearer representation of follicle halves. After review of neighboring tissue sections, the previously chosen section appears to be cut through primarily the ruptured side of the follicle wall. The newly selected follicle is more centrally located within the follicle.

P-adj: 5.72E-05 P-adj: 5.33E-17 P-adj: 7.95E-11 P-adj: 1.85E-14

10 Unruptured (no hCG)
Ruptured (no hCG)
log2[TPM + 1]

Unruptured (post-hCG)
5 Ruptured (post-hCG)

0

-5
Fxyd1 Nap1l5 Serpinb2 Gas1

B
In vivo Fxyd1 Ex vivo
C
In vivo Nap1l5 Ex vivo
No hCG

D Serpinb2 E Gas1 Ex vivo
In vivo Ex vivo In vivo
Post-hCG

REVISED Figure 4. Asymmetric gene expression was validated using RNAscope in situ hybridization assays and was consistent between in vivo and ex vivo follicles.

Comment 3:
Line 245-246 (related to the previous comment): For in vivo validation of asymmetric patterns, the text states that the putative unruptured side (light pink arrow and box) is selected opposite to the thinning region of the ruptured side (light blue arrow and box). However, that is not the case in images with Fxyd1 and Gas1 staining in Fig 4B & 4E.
Thank you for this note. We have repositioned the arrows to match the approximate location of the boxes.

Comment 4:
Lines 265-300: Gene ontology analysis of genes enriched in ruptured or unruptured walls revealed several novel asymmetric pathways. Visualization of these pathway activities in the in vivo system would really strengthen the paper. Using the genes associated with these pathways, the authors should estimate and visualize pathway module scores on spatial transcriptomic datasets from ovulating ovaries published recently (PMID: 38826447 and PMID: 37662215). The authors could additionally select apex and the base (putative ruptured vs unruptured sites) of growing and preovulatory follicles and do a quantitative comparison of the module scores. This additional analysis could significantly strengthen the novel asymmetric pathway results and would be of value to the field.

Thank you for this suggestion. We agree that there would be great value to visualizing asymmetry of follicles using existing spatial transcriptomics data. We attempted this, but unfortunately encountered limitations to completing this analysis. In our RNAscope analysis, we were able to select sections that best captured both the ruptured and unruptured side of the follicle wall by looking at adjacent sections so that we could assess the overall structure of the follicle that the section was taken from. In spatial transcriptomics datasets, however, only a small number of sections are used for analyses and these are not always optimized for selection of features, like the presence of many follicles since you are relying on a 2D slice of a tissue. From the available published data, it is impossible to tell what position the follicle is in within the section. Therefore, only a small number of follicles, if any, within these datasets are relevant for

demonstrating asymmetry.

Despite this, we did attempt to perform module scoring using differentially expressed genes from our dataset as well as show visualization of individual genes from pathway analysis. We performed this analysis with the two datasets suggested, where the Huang et al., data is true single-cell resolution and the Mantri et al., data is spot-based resolution, and both provide temporal resolution. As expected, these results do not clearly demonstrate asymmetry (see below). We do not believe that this is because the results to do not hold in vivo, rather, the available published datasets do not have the follicle numbers and orientation to really demonstrate this. For these reasons, we have decided not to integrate this data into our manuscript. We have incorporated a line into our discussion proposing 3D imaging techniques and spatial transcriptomics as potential future directions for more detailed study of asymmetric molecular processes.

Line 557-559: Future studies incorporating spatial transcriptomics or whole-ovary immunofluorescence with tissue clearing may provide a higher degree of spatial resolution than was possible with our technique[135,180-182].

A B

C D

E F

Response to reviewer Figure 1: A) Module scores for the putative ruptured side and B) in an ovary at time point 0h using data from PMID 38826447. C) Module scores for the putative ruptured side and D) in an ovary at time point using data from PMID 37662215. E) Localization of Nap1l5 using data from PMID 38826447 and F) from PMID 37662215.

Comment 5:
Lines 265-300 (Related to the previous comment): Were the theca cells retained in the follicles during isolation for ex vivo experiments? It is not very clear if granulosa cells or theca cells are driving some of these asymmetric molecular patterns in the follicle wall. Asymmetry in several pathways, such as angiogenesis, muscle contraction, apoptosis, and even in signaling pathways such as NOTCH, WNT, and BMP, could be attributed to granulosa cells alone, theca cells alone, or even both. The authors can address this limitation of the technique by analyzing high-resolution spatial transcriptomics data sets discussed in the previous comment. Identification of the cell types for these functional pathways would not only strengthen this work but would influence thinking in the field on how to use these discoveries for reproductive medicine.

Thank you for this suggestion. In this manuscript, we used an enzymatic isolation technique that results in breakdown of the basement membrane of follicles and loss of the majority of the theca cell layer. When these follicles are cultured, previous data from our group shows that the granulosa cells differentiate into theca-like cells at the periphery[1]. Since we also remove the cumulus-oocyte-complex from our microdissected follicle sides, the sequenced samples should consist of mostly granulosa cells and theca cells.

Citations:
1. Babayev, E., Xu, M., Shea, L. D., Woodruff, T. K., and Duncan, F. E.. Follicle isolation methods reveal plasticity of granulosa cell steroidogenic capacity during mouse in vitro follicle growth. Molecular human reproduction 28(10), (2022).

We added the following text to clarify this point:

Line 381-386: In our system, the enzymatic isolation process breaks down the follicle basement membrane causing loss of much of the theca layer[136]. As follicles are grown in culture, they recover a theca layer that is hormonally active and grows throughout culture [136]. Since the COC was removed and discarded during microdissection, we expect that the sequenced samples consist primarily of granulosa and theca cells.

To better understand which cell types are driving each of the highlighted signaling pathways, we integrated results from our previously published single cell dataset into a new figure of the manuscript, and we are grateful to the reviewer for this suggestion which has provided a further dimension to our findings.
Figure 8. Analysis of published single-cell transcriptomic datasets reveals cell type specificity of top enriched pathways
Cell expression analysis derived from Huang et al., 2024 identified cell type specificity for A) genes enriched in the unruptured side and B) genes enriched in the ruptured side.

Line 751-757:
Cell Ranger was used for demultiplexing after acquiring the raw sequencing data as well as the subsequential alignment187. Seurat 4.2 was used in R 4.2.2 was used for initial quality filtering which removed cells with greater than 20% mitochondrial gene expression or less than 3000 expression counts188-192. The dataset then went through scaling, normalization, dimension reduction, doublet removal, and clustering, which is how granulosa and theca cell clusters across the time course of ovulation are subsetted. The heatmap shows the expression of those genes in this single-cell dataset.
Line 378-406:
Analysis of published single-cell transcriptomic datasets reveals cell type specificity of top enriched pathways
To refine the cell types that drive individual asymmetric pathways within ovulatory follicles, we assessed gene expression patterns from published single-cell sequencing data135. In our system, the enzymatic isolation process breaks down the follicle basement membrane causing loss of much of the theca layer136. As follicles are grown in culture, they recover a theca layer that is hormonally active and grows throughout culture136. Since the COC was removed and discarded during microdissection, we expect that the sequenced samples consist primarily of granulosa and theca cells. To clarify whether key enriched pathways are driven by theca cells, granulosa cells, or both, we assessed expression of several DEGs in theca and granulosa clusters from a single cell sequencing dataset generated across the time course of ovulation (Figure 8). Specifically, we analyzed expression of these genes in three identified granulosa cell clusters (granulosa 1, 2, and 3) and two identified theca cell clusters (theca 1 and 2).
On the unruptured side, we find that genes related to smooth muscle contraction and vascular remodeling are largely enriched in the theca layer, which is consistent with known ovarian biology (Figure 8A). Genes related to inhibition of proteolysis, Wnt signaling, and metal ion signaling are also generally enriched in theca cells (Figure 8A). IL-11 signaling exhibits mixed theca and granulosa cell expression, with Jak1 enriched in the granulosa 1 cluster and Stat3 and IL11 both enriched in the theca 2 cluster (Figure 8A). Genes related to glycolysis are enriched in the granulosa 1 cluster, consistent with the known central role of granulosa cells in metabolic function of follicles in response to ovulatory hormonal stimulation (Figure 8A).
On the ruptured side, oxidative stress related genes were enriched across all three granulosa clusters (Figure 8B). ECM remodeling had mixed expression between granulosa and theca cell types, suggesting that remodeling is occurring in both cell types (Figure 8B). prostaglandin signaling, BMP signaling, and amino acid transport all had mixed expression patterns, which may indicate communication between theca and granulosa cells during the ovulatory period (Figure 8B). Jag-Notch signaling, while lowly expressed, was largely restricted to the theca layer (Figure 8B). This analysis further refined our pathway results to identify which cell types may participate in each of these key pathways.

Lines 1482-1490:
Figure 9. Summary of Key findings.
Distinct regions of the ex vivo follicle wall exhibit functional and molecular differences that both recapitulate known ovulation biology and represent novel pathways that may participate in asymmetric functions during the periovulatory period. These pathways, identified through gene ontology, represent areas of potential biological significance for the processes of ovulation and luteinization. Further analysis identified cell-type specificity of each pathway. Some pathways were enriched primarily in granulosa cells (green), theca cells (orange), or a combination of both cell types (gray). The cumulus-oocyte-complex, which was excluded from our analysis, is shown in black and white.

Added to discussion:

Lines 453-455: Using single cell data, we found that the genes driving both vascular remodeling and smooth muscle related pathways were localized to theca cells. This is consistent with known in vivo localization of both peri-follicular vasculature and smooth muscle function during ovulation12.

Lines 464-467: Our analysis also demonstrated localization of key protease inhibitors to theca cell clusters. This is consistent with prior studies showing localization to the theca in vivo and may directly support luteal formation31.

Lines 473-476: Our single cell analysis showed, as expected, that glycolytic processes were largely restricted to granulosa cell clusters124,144,145. Genes involved in Wnt signaling pathways were largely restricted to theca cell clusters, which has not previously been reported.

Lines 485-486: Mt1 expression has also been shown to be localized to the theca layer during the ovulatory period, consistent with our single cell analysis156.

Lines 495-498: Our cell type specificity analysis suggests that IL-11 signaling may be facilitated by communication between granulosa and theca cell layers based on expression of Jak1 in granulosa clusters and expression of Il11 and Stat3 in theca clusters.

Lines 507-509: Further, our analysis suggests that oxidative stress processes may be primarily localized to the granulosa cell layers and may play an important role in follicle wall thinning leading up to follicle rupture.

Lines 523-524: Our analysis suggests that both theca and granulosa cells may participate in amino acid transport, with specific solute carrier channels localized to each cell type.

Lines 530-531: In our cell type analysis, we found low expression of Jag1 and Notch2 across both theca and granulosa clusters.

Lines 571-573: Finally, we harnessed a published single cell dataset to identify cell-type specificity of each of these asymmetric pathways to further elucidate their potential function in the periovulatory period (Figure 9).

Comment 6:
Lines 528-529: Were secondary follicles collected from multiple animals for every ex vivo experiment described in the manuscript? Details regarding how many animals were used should be included in future captions and/or the methods section.

We have added sample size details to all figure legends and manuscript text.

Version 1:

Reviewer comments:

Reviewer #2

(Remarks to the Author)
The authors have addressed previous concerns.
This is an outstanding contribution to our knowledge of the ovulatory process.

Reviewer #3

(Remarks to the Author)
The authors had performed a thorough analysis to uncover several novel insights into follicle-inherent, spatially defined markers and pathways during follicle rupture. The figures are clear, easy to follow, and visually compelling. I also appreciated the supplemental data provided.

The authors now have addressed all my comments. I greatly value the updated in vivo images and the new analysis of cell-type specificity of rupture-associated programs. This additional analysis adds significant value and detail to the work.

I recommend that this paper be accepted for publication.

Open Access This Peer Review File is licensed under a Creative Commons Attribution 4.0 International License, which permits use, sharing, adaptation, distribution and reproduction in any medium or format, as long as you give appropriate credit to the original author(s) and the source, provide a link to the Creative Commons license, and indicate if changes were made.
In cases where reviewers are anonymous, credit should be given to 'Anonymous Referee' and the source.
The images or other third party material in this Peer Review File are included in the article's Creative Commons license, unless indicated otherwise in a credit line to the material. If material is not included in the article's Creative Commons license and your intended use is not permitted by statutory regulation or exceeds the permitted use, you will need to obtain permission directly from the copyright holder.
To view a copy of this license, visit https://creativecommons.org/licenses/by/4.0/
